# Nanomedicine Meets Immunotherapy: Advancing Adoptive Cell Therapy with Nanoparticles in the Treatment of Cancer with Sustainability Perspectives

**DOI:** 10.1002/advs.202522864

**Published:** 2026-03-07

**Authors:** Erica Frostegård, Tatiana Bobrova, Amalie Leinebø, Thibault Leray, Shayamita Ghosh, Lorena García‐Hevia, Chiara Puccinelli, Lorenzo Riccio, Jacopo Sorani, Davide Bonifazi, Julien Caumartin, Emmanuel Donnadieu, Florence Gazeau, Roland Hischier, Else Marit Inderberg, Mónica L. Fanarraga, Maria Loustau, Bernd Nowack, Sébastien Wälchli, Cécilia Ménard‐Moyon

**Affiliations:** ^1^ CNRS, Immunology Immunopathology and Therapeutic Chemistry UPR 3572 University of Strasbourg Strasbourg France; ^2^ INSERM UMR 1186 Integrative Tumor Immunology and Immunotherapy Gustave Roussy, Fac. de Médecine‐University Paris‐Sud Université Paris‐Saclay Villejuif France; ^3^ University of Paris City CNRS Inserm NABI Paris France; ^4^ Translational Research Unit Section for Cellular Therapy Department of Oncology Oslo University Hospital Oslo Norway; ^5^ Medical Faculty University of Oslo Oslo Norway; ^6^ The Nanomedicine Group Universidad de Cantabria‐Instituto de Investigación Valdecilla‐IDIVAL Avda Herrera Oria s/n Santander Spain; ^7^ Institute of Organic Chemistry, Faculty of Chemistry University of Vienna Vienna Austria; ^8^ Swiss Federal Laboratories for Materials Science and Technology (Empa) Technology and Society Laboratory St. Gallen Switzerland; ^9^ Exosiris Le Vésinet France; ^10^ University of Strasbourg Institute of Advanced Study (USIAS) Strasbourg France

**Keywords:** chimeric antigen receptor, drug delivery, immunomodulator, magnetic hyperthermia, photothermal therapy

## Abstract

Immunotherapy has achieved remarkable clinical success in certain cancers, particularly through adoptive cell therapy (ACT), where T cell engineering with chimeric antigen receptor (CAR) has driven major clinical breakthroughs in the treatment of hematologic malignancies. However, efficacy against solid tumors remains limited due to multiple barriers, including the scarcity of tumor‐specific antigens, antigen heterogeneity, immunosuppressive tumor microenvironment, and physical obstruction of T cell infiltration by dense extracellular matrix. Nanoparticle (NP)‐based approaches can overcome these obstacles and enhance ACT by improving tumor immunogenicity and vascular permeability, while reducing off‐target toxicity to healthy tissues. This review discusses different strategies leveraging NPs to enhance ACT, including the delivery of immunomodulators and chemotherapeutics, NP‐mediated hyperthermia, magnetic guidance to improve T cell accumulation in tumors, and in vivo NP‐mediated generation and activation of CAR T cells. While prioritizing patient safety is essential, it does not fully reflect the range of risks and challenges associated with novel nanomedicines. In this regard, we discuss how integrating environmental impact assessments early in the development process is crucial for identifying key impact areas of concern and steering innovation towards more sustainable and responsible designs. Finally, we also identify current challenges, and discuss potential solutions and future research directions, including safety and sustainability.

## Graphical abstract

This review surveys nanoparticle‐based strategies to enhance adoptive cell therapy, particularly CAR‐T cell approaches, in solid tumor treatment. It describes how nanoparticles can improve tumor immunogenicity and T‐cell infiltration while reducing toxicity, and how they enable in vivo CAR‐T cell generation. It also addresses safety considerations, translational challenges, environmental impact, and future directions towards effective and sustainable nanomedicine‐enabled immunotherapies.

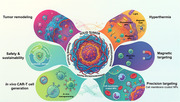

## 1 Introduction

### 1.1 Adoptive Cell Therapy for Cancer Treatment

The goal of cancer immunotherapy is to modify the immune response of the patient to favor the elimination of cancer cells. Among the diverse range of immunotherapeutic strategies, immune checkpoint inhibitors (ICIs) and adoptive cell therapy (ACT) have revolutionized the field of immuno‐oncology. ACT involves the intravenous infusion of immune cells—either tumor‐infiltrating lymphocytes or peripheral blood lymphocytes—that may be genetically modified to enhance their antitumor activity. ACT encompasses three main approaches: (1) tumor‐infiltrating lymphocyte therapy, (2) T cell receptor (TCR)‐engineered T cells, and (3) chimeric antigen receptor (CAR) T cells, with newer adaptations including CAR natural killer (NK) cells and CAR‐macrophages under early investigation [1]. The first proof‐of‐concept for ACT was demonstrated by Steven Rosenberg and colleagues in 1988, when they showed that tumor‐infiltrating lymphocytes expanded ex vivo and reinfused into patients, together with high‐dose interleukin‐2 (IL‐2), could mediate tumor regression in metastatic melanoma [2]. Over the following decades, multiple phase I and II trials in melanoma and other cancers refined tumor‐infiltrating lymphocyte manufacturing, conditioning regimens, and patient selection, laying the foundation for later pivotal studies [3, 4]. In 2024, the Food and Drug Administration (FDA) granted an accelerated approval to lifileucel, a tumor‐infiltrating lymphocyte‐based therapy for metastatic melanoma, following a successful phase II trial [5]. Tumor‐infiltrating lymphocyte therapy involves isolating tumor‐resident T cells from surgically resected tumors, followed by ex vivo expansion [6]. Although lifileucel is currently approved only for metastatic cutaneous melanoma, tumor‐infiltrating lymphocytes can be successfully generated from other solid tumors. However, several limitations hinder broader application, including difficulties accessing tumor tissue, failure to expand tumor‐infiltrating lymphocytes in culture, and logistical complexities requiring specialized clinical infrastructure [7].

Another major advancement in ACT is the development of CAR T cells. In contrast to tumor‐infiltrating lymphocytes, CAR‐T cell therapy uses peripheral blood T cells that are genetically engineered ex vivo to express synthetic receptors that recognize tumor antigens directly, without the need to match the human leukocyte antigen (HLA) type of the patient [8]. These chimeric receptors combine extracellular antigen‐binding domains with intracellular signaling and costimulatory domains, first conceptualized by Zelig Eshhar [9]. Four generations of CAR designs have since been developed. First‐generation CARs, containing only the CD3ζ signaling domain, showed limited clinical activity. Second‐generation CARs incorporated a costimulatory domain (CD28 or 4‐1BB), while third‐generation CARs included both domains. Finally, fourth‐generation CARs added inducible cytokine production [10]. To date, the FDA has approved several CAR‐T cell therapies for hematological malignancies, including B cell acute lymphoblastic leukemia, B cell lymphoma, and multiple myeloma. In solid tumors, results of clinical trials have been more limited, showing variable efficacy and highlighting major challenges such as: (1) lack of tumor‐specific antigens and “on‐target”/“off‐tumor” toxicity; (2) tumor antigen heterogeneity, where variable or loss of antigen expression allows tumor escape; (3) poor trafficking to tumor sites due to mismatched chemokine‐chemokine receptor profiles and physical barriers; (4) limited in vivo persistence, where transferred T cells fail to survive or expand due to insufficient cytokine support and an immunosuppressive tumor milieu; and (5) functional exhaustion, marked by chronic antigen stimulation leading to diminished effector function and upregulation of inhibitory receptors [11].

Genetically engineered TCR‐T cell therapy has also demonstrated therapeutic potential. Unlike CAR T cells, which target surface antigens, TCR T cells are designed to recognize intracellular antigens presented via the major histocompatibility complex (MHC). This allows TCR T cells to access a broader repertoire of tumor‐associated antigens. These therapies involve the genetic modification of patient‐derived T cells to express a specific TCR, typically isolated from high‐affinity clones [12, 13]. Importantly, TCR T cells require lower antigen density and lower affinity to trigger T cell activation compared to CAR T cells. Recent studies suggested that TCRs with low to intermediate avidity may be more effective in tumor control, while high‐avidity TCRs may increase the risk of autoimmunity [14, 15]. In 2024, the FDA granted accelerated approval to afamitresgene autoleucel (TECELTA, Adaptimmune LLC), the first TCR‐T cell therapy for solid tumors [16, 17]. This treatment targets MAGE‐A4, a melanoma‐associated antigen expressed in various solid cancers, and is indicated for adults with unresectable or metastatic synovial sarcoma. Patients receive chemotherapy (doxorubicin and/or ifosfamide) and a lymphodepleting regimen (fludarabine and cyclophosphamide) prior to T cell infusion. While TCR T cells offer certain biological advantages, research and clinical development have focused on CAR T cells. TCR T cells are restricted by HLA type and target only peptide antigens presented on HLA molecules, which limits their universality, while CARs can recognize surface antigens in an HLA‐independent manner, making them applicable to a broader patient population. Moreover, early CAR‐T cell successes in B cell malignancies have spurred significant investment and a larger number of clinical trials compared to TCR‐T cell approaches. Other challenges associated with TCR‐T cell therapy include complex and labor‐intensive TCR epitope identification, risks of on‐target/off‐tumor toxicities due to antigen expression in healthy tissues, and immune escape via downregulation of MHC cell‐surface expression, which can occur through tumor‐driven genetic mutations, epigenetic silencing, or alterations in antigen processing machinery, thereby preventing TCR recognition [18, 19].

CAR T cells can be very toxic with cytokine release syndrome, neurotoxicity, immune cell‐associated neurotoxicity syndrome, and other immune‐related adverse events, which may require intensive monitoring and prompt management with immunosuppressive agents such as corticosteroids or anti‐IL‐6 therapies. This may be explained by the fact that CAR T cells recognize abundant, non‐MHC‐restricted antigens and can therefore trigger rapid and robust activation, whereas TCR T cells and tumor‐infiltrating lymphocytes rely on HLA‐restricted antigen presentation, which is often less uniformly expressed and may result in slower or less intense activation. In addition to systemic toxicities, CAR T cells can cause on‐target, off‐tumor effects when the targeted antigen is also expressed at low levels on healthy tissues, potentially leading to serious organ damage, such as pulmonary, cardiac, or hepatic toxicity, depending on the antigen distribution.

ACT has already shown great promise in both hematologic and solid tumors. However, the current state of treating solid tumors presents significant barriers, including the need for complex ex vivo cell manufacturing, risks of immune‐related toxicities, and reduced efficacy due to the reasons described above [1]. To overcome these challenges, a wide range of combinatorial strategies is currently under investigation. A recent comprehensive review summarized ongoing clinical trials combining CAR‐T cell and tumor‐infiltrating lymphocyte therapies with various agents, including ICIs, tumor‐targeting antibodies, bispecific T cell engagers (BiTEs), small molecules (e.g., tyrosine kinase inhibitors such as ibrutinib), protein degraders (e.g., lenalidomide), and oncolytic viruses [20].

In solid tumors, the tumor microenvironment (TME) is a major barrier to ACT efficacy [21, 22]. This hostile milieu, characterized by physical barriers, immunosuppressive cells, inhibitory cytokines, and metabolic stress, impedes T cell trafficking and infiltration, suppresses cytotoxic function, and shortens persistence. Dense and aberrant extracellular matrix (ECM), high interstitial fluid pressure, and abnormal vasculature physically obstruct the infiltration and distribution of ACT, while strong immunosuppressive mechanisms further dampen antitumor activity. To overcome these limitations, numerous approaches have been developed to modulate the TME and restore antitumor immune function [23]. These include the use of CAR T cells engineered to secrete proinflammatory cytokines (e.g., IL‐12) [24], to resist inhibitory signals (e.g., dominant‐negative tumor growth factor β (TGF‐β) receptors), or to express chemokine receptors favoring tumor homing [25]. Additional strategies aim to modulate the TME to enhance immunogenicity, such as the use of Toll‐like receptor (TLR) agonists, which activate innate immunity and promote effector T cell recruitment, and stimulator of interferon genes (STING) pathway agonists, which induce type I interferon responses and chemokine production, facilitating immune cell infiltration into tumors. To address tumor heterogeneity, multimodal targeting combinations integrating CARs and TCRs within the same cell are under development [19]. Finally, novel NP‐based delivery systems are being investigated to improve T cell infiltration into tumor sites. These innovative approaches will be further discussed in this review [10, 20].

Beyond the TME, tumor cell intrinsic features can reduce CAR‐T cell activity due to sensitivity to cytotoxic mechanisms, cytokine responsiveness, and expression of adhesion molecules, including perforin/granzyme‐mediated cytolysis, impaired responsiveness to pro‐inflammatory cytokines such as interferon‐γ (IFN‐γ), and downregulation or loss of adhesion molecules (e.g., ICAM‐1, VCAM‐1) required for stable immunological synapse formation. Defects in apoptotic pathways, such as mutations in Fas‐associating death domain‐containing protein or caspases, can also reduce tumor cell susceptibility to CAR‐T‐mediated killing [26]. Finally, tumor response to IFN‐γ is particularly important, as IFN‐γ can upregulate MHC molecules and adhesion proteins, such as ICAM‐1, promoting effective CAR‐T engagement [27, 28].

Although tumor‐infiltrating lymphocyte, TCR‐T, and CAR‐T cell therapies differ in their sources, antigen recognition strategies, and manufacturing processes, they share a common goal: generating durable tumor‐specific T cell responses in solid tumors. However, all three modalities face similar limitations. Indeed, poor immune cell infiltration, tumor‐mediated immunosuppression, and the complexity of ex vivo cell engineering restrict the clinical use of ACT to treat solid tumors. Efforts to overcome these barriers through systemic cytokine or immunomodulator administration are restricted by dose‐limiting toxicities and limited spatial control. These challenges underscore the need for localized and sustained modulation of T cell function and the TME. NPs can address these needs by enabling in vivo CAR expression, enhancing tumor targeting with lower systemic toxicity, allowing tumor‐selective immunomodulation, and providing localized cytokine modulation. NPs have also been used to alter the physical and immunosuppressive barriers by hyperthermia, and to deliver CAR mRNA, cytokines, and immunomodulators, leading to better therapeutic outcomes.

### 1.2 Nanoparticles in Cancer Therapy

NPs can generally be classified as inorganic and organic, with inorganic spanning metallic, ceramic, and carbon‐based nanomaterials, and organic including polymeric and lipid NPs (LNPs). Metallic NPs (e.g., gold NPs) and carbon nanomaterials can convert light into heat, which is the basis for photothermal therapy (PTT). They absorb light in the biological window (650‐1350 nm), the range of wavelengths where tissue absorption and scattering of light is minimal, thus the penetration of light is maximized [29]. In magnetic hyperthermia (MH) therapy, magnetic NPs, such as iron oxide NPs, generate heat under an alternating magnetic field [30]. The heat generated by PTT or MH aims to kill or damage the targeted cancer cells. In contrast to light irradiation, there is no penetration depth limitation of a magnetic field, making MH better suited for deep‐seated tumors. Iron oxide and/or gold NPs have been exploited for hyperthermia to reverse the physical and immunological barriers of the TME. Iron oxide NPs present many advantages, not only for hyperthermia, but also as a contrast agent for magnetic resonance imaging (MRI) and for tumor targeting under an external magnetic field. Many polymeric NPs, such as poly(lactic‐co‐glycolic) acid (PLGA) NPs, are biocompatible, biodegradable, and have a high loading capacity, making them excellent drug delivery candidates [31]. Likewise, LNPs are biodegradable and are widely studied for drug delivery as well as gene delivery [32]. Both polymeric NPs and LNPs complexed to plasmid DNA encoding a specific CAR have been used to efficiently transduce T cells in vivo. In recent years, a significant increase (38%) in nanomedical clinical trials has been observed, reflecting growing technological maturity and momentum towards clinical translation [33]. Oncology continues to dominate in clinical trials [34], accounting for approximately 30% of trials, underscoring the prioritization of nanomedicine for high‐mortality diseases [33]. However, despite these advances, studies involving nanomedicines still represent only about 0.8% of all registered clinical trials, highlighting translational barriers related to regulatory complexity and high manufacturing costs [33]. Notably, there is a clear shift in nanomedicines, with increasing use of micelles, polymeric, and metallic NPs compared to the traditional liposomes [35]. The first nanomedicine to be approved by the FDA for cancer treatment was Doxil in 1995, consisting of doxorubicin encapsulated in polyethylene glycol (PEG)‐functionalized liposomes [36]. Protein‐based NPs have also been approved, such as Abraxane in 2005 [37]. Remarkably, iron oxide NPs named NanoTherm have been approved for MH to treat glioblastoma [38]. Currently, silica NPs coated with a gold shell (named AuroShell) are investigated in clinical trials for PTT for the treatment of different types of cancer [39]. In the context of cancer immunotherapy, several clinical trials have investigated the combination of organic NP‐based therapies with immunotherapies, many of which show promising outcomes [40].

The capacity of NPs to accumulate in tumors is often attributed to the enhanced permeability and retention (EPR) effect, which is due to leaky vasculature and impaired lymphatic drainage in tumors. However, this passive targeting mechanism has been increasingly debated [41], and recent studies showed that NPs enter tumors through active processes via endothelial cells [42]. In contrast to passive targeting, active targeting employs surface functionalization of NPs to recognize molecular markers expressed on tumor cells or in the TME. This strategy improves the targeting specificity, and it can increase cellular uptake by receptor‐mediated endocytosis [43]. Commonly used ligands for active targeting include antibodies, antibody fragments, peptides, and small molecules.

The physicochemical characteristics of NPs, such as their size, shape, and charge, can be tuned to favor their tumor accumulation [44]. Because of the high complexity and large variations between different NP systems, it is hard to define universally optimal values for these parameters. Nevertheless, some principles are widely accepted. For the NPs to reach the tumor, they need to have a long blood circulation time. Small NPs (<6 nm) are rapidly eliminated through the kidneys, and thus have a short circulation time [45]. On the other hand, as the NP size increases, their tissue penetration decreases [46]. Thus, sizes between 6 and 200 nm are generally considered optimal for tumor accumulation. Another crucial parameter is the zeta potential of the NPs. It has been observed that positively charged NPs cause cytotoxicity due to electrostatic interactions with the negatively charged cell membranes. In contrast, strongly negatively charged NPs are rapidly cleared by the reticuloendothelial system [47]. Thus, weakly charged NPs are usually considered optimal.

Due to the limitations of ACT for solid tumors, several studies are now focusing on combining ACT with other therapies, such as chemotherapy, radiotherapy, oncolytic viruses, and ICIs [48]. Additionally, NPs have emerged as a promising co‐therapy to enhance ACT [49, 50, 51]. Beyond their use as efficient vectors for gene transduction for therapeutic T cell engineering [52], they can be utilized to pretreat the TME before ACT [53]. Indeed, by simultaneously targeting several barriers of the TME, including immune suppression [54], poor infiltration [55]. and physical stromal resistance [56], multifunctional NPs offer a synergistic strategy to enhance ACT, especially by increasing T cell infiltration and persistence, remodeling the TME, and supporting T cell‐mediated tumor eradication [57]. Moreover, NPs can be designed not only to accumulate in the tumor, but also to release drugs specifically inside the tumor. This can be achieved either by exploiting certain characteristics of the TME, including its acidity [58], or by using external stimulation such as laser irradiation or ultrasound [59].

**FIGURE 1:**
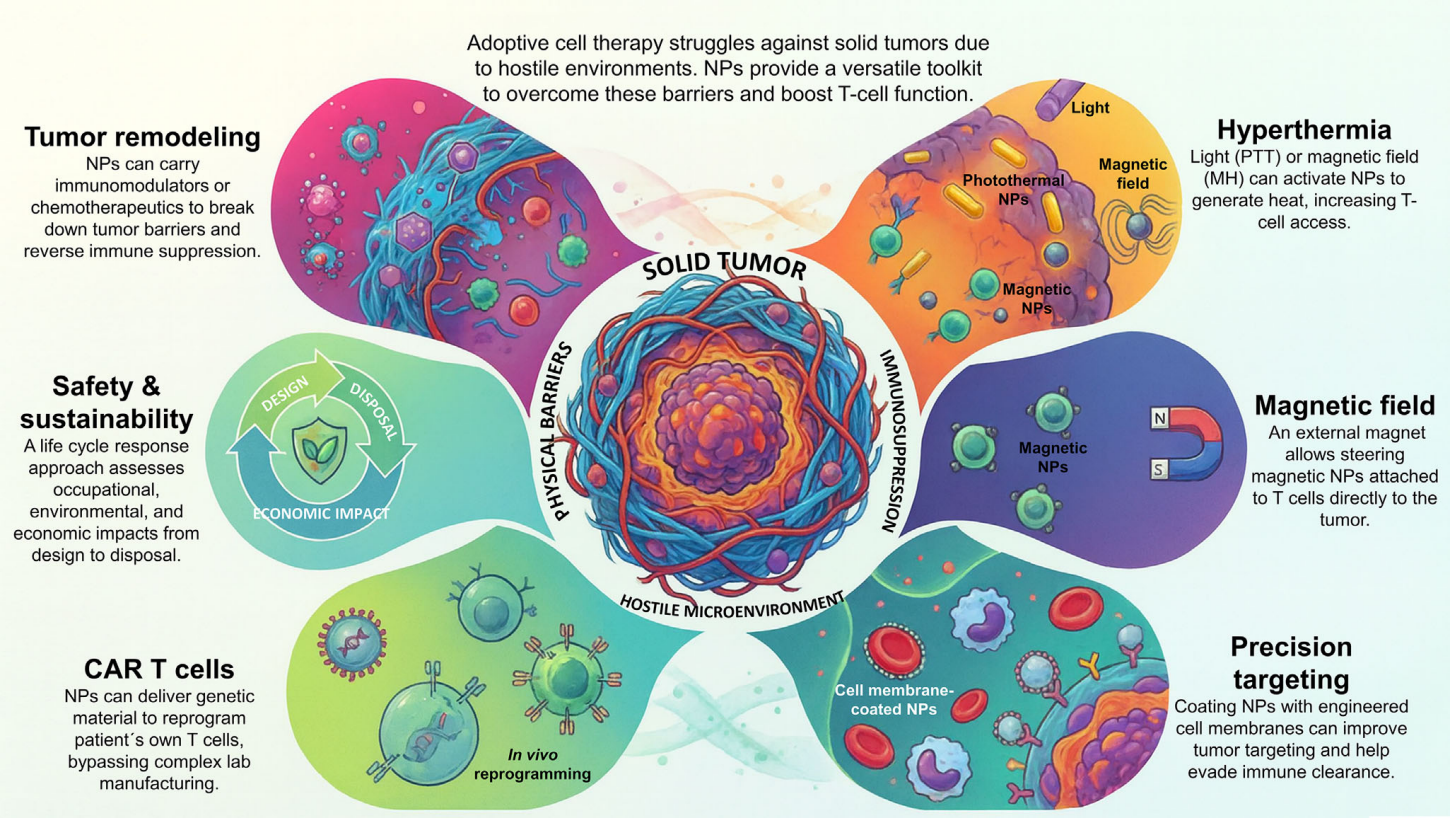
Overview of the different strategies covered in the review for advancing ACT with NPs in cancer treatment. This figure was generated with the help of NotebookLM.

In this review, we discuss how NP‐mediated drug delivery can enhance ACT by improving T cell persistence, expansion, and functionality within the hostile TME, in particular for the treatment of solid tumors (Figure 1). We then examine combination approaches, such as NP‐mediated hyperthermia and ACT, to sensitize tumors and enhance therapeutic efficacy. Emerging approaches, such as magnetically targeted CAR T cells and in vivo NP‐facilitated CAR‐T cell generation and controlled activation, are presented as innovative solutions to overcome current limitations in the delivery and manufacturing of CAR T cells. Furthermore, we describe the use of NPs coated with the membrane of engineered cells to improve targeting precision and reduce off‐target effects. Beyond these advancements, the entire life cycle of nanomedicines, which encompasses a range of challenges linked to occupational health and environmental impact, should be considered. From laboratory synthesis and large‐scale manufacturing to clinical use, patient excretion, and eventual environmental release, significant issues and measurable effects on both occupational health and the environment can arise at every stage. In this context, we discuss key challenges related to occupational exposure, environmental release and transformation, ecotoxicity, and sustainability of nanomedicines. Together, these sections provide a comprehensive overview of how nanotechnology can be leveraged to optimize ACT and pave the way for next‐generation safe and sustainable cancer immunotherapies.

## 2 Enhancing Adoptive Cell Therapy with Nanoparticle‐Mediated Drug Delivery

This section reports different drug delivery strategies using NPs as nanocarriers to enhance ACT through (i) overcoming the physical barriers of solid tumors, (ii) reversing the immunosuppressive environment of the tumor via the delivery of immunomodulators, and (iii) combination with chemotherapy.

### 2.1 Nanoparticles for Overcoming the Physical Barriers of Solid Tumors

The complex TME imposes both physical and immunological barriers to T cell functions [21, 60, 61]. In solid tumors, the microenvironment is composed of tumor cells, stromal cells (including fibroblasts and endothelial cells), immune cells, the ECM, abnormal vasculature, various signaling molecules (such as chemokines and cytokines), and a metabolically stressed environment characterized by hypoxia, low pH, and high interstitial fluid pressure. One of the principal physical barriers to CAR‐T cell infiltration is the ECM, a dense network of proteins, such as collagen and fibronectin, which restricts the movement and penetration of immune cells within the tumor core. Tumor‐associated fibroblasts, activated by factors, such as TGF‐β, further reinforce this barrier by producing a fibrotic, stiff stroma that limits immune cell access. In addition to its role in stromal remodeling, TGF‐β signaling is also a key mechanism of immune suppression in the TME, contributing to the overall resistance to CAR‐T cell therapy [62]. An abnormal tumor vasculature also plays a crucial role in hindering T cell trafficking [63]. Unlike normal blood vessels, tumor vasculature is often disorganized and leaky, with reduced expression of adhesion molecules that are essential for T cell extravasation [64, 65]. This vascular dysfunction impairs the efficient homing of CAR T cells to tumor sites. In addition, the chemokine milieu within solid tumors is often unfavorable for effector T cell recruitment, instead favoring the accumulation of immunosuppressive populations such as regulatory T cells (Tregs) and myeloid‐derived suppressor cells [66]. This occurs because the chemokines secreted by the tumor do not match the chemokine receptors expressed on CAR T cells, creating a chemokine‐receptor mismatch that reduces the likelihood of CAR T cells successfully locating and infiltrating tumor tissue. In addition, chemokine‐mediated retention mechanisms contribute to T cell exclusion. Cancer‐associated fibroblasts secrete CXCL12, which binds CXCR4 on T cells and retains them in perivascular regions, preventing migration into the tumor core. The CXCL12/CXCR4 axis is a major driver of immune exclusion, acting together with ECM deposition and abnormal vasculature to limit CAR‐T cell infiltration [67, 68] It also promotes regulatory T cell dominance over cytotoxic T cells, reinforcing immunosuppression [69]. This has been validated in canine tumors, where cancer‐associated fibroblast‐derived CXCL12 modulated by TGFβ1 regulated T cell dynamics [69], and in human esophageal squamous carcinoma, where fibroblast activation protein‐positive fibroblasts enhanced tumor progression and immunoregulation via CXCL12/CXCR4 [70]. These findings highlight the therapeutic potential of CXCR4 blockade, CXCL12 neutralization, and cancer‐associated fibroblast‐targeting strategies to improve ACT efficacy [71, 72].

Finally, metabolic features of the TME, such as elevated lactic acid levels, low glucose availability, and hypoxia, not only contribute to the acidic microenvironment but also impair T cell survival, differentiation, and cytotoxic activity, further limiting the therapeutic potential of CAR T cells in solid tumors [73, 74].

To overcome these challenges, nanotechnology may play a key role. In fact, clinically approved NPs are capable of selectively being accumulated in tumors, enhancing drug delivery, and being safely degraded and/or cleared with minimal toxicity [34, 35, 75]. As a result, an increasing number of studies have focused on leveraging NPs to disrupt the physical barriers characteristic of solid tumors. These NP‐based strategies are aimed at enhancing CAR‐T cell infiltration by targeting and remodeling the ECM, promoting the normalization of tumor vasculature, or delivering therapeutic agents that modulate the tumor stroma. In fact, several NPs have been engineered to actively degrade the dense tumoral ECM using matrix‐modifying enzymes. Nanocarriers loaded with hyaluronidase or collagenase can locally digest hyaluronic acid‐ and collagen‐rich stromal components, thereby reducing interstitial fluid pressure and loosening the compact ECM network [76]. thereby facilitating deeper penetration of therapeutic agents into solid tumors [77]. This enzymatic remodeling creates permissive pathways within the tumor mass that allow bulky CAR T cells to infiltrate the tumor core rather than remaining confined to perivascular regions [78]. Importantly, enzyme‐loaded NPs improve intratumoral distribution of adoptively transferred T cells and enhance their antitumor efficacy while limiting systemic toxicity compared to free enzyme administration [79].

Beyond these representative cases, additional NP‐enabled strategies have been reported, including ECM remodeling with lipoxygenase inhibitors or cancer‐associated fibroblast‐targeted siRNA [78, 80]. vascular normalization via vascular endothelial growth factor‐blocking nanocarriers [81], stromal depletion through fibroblast activation protein‐targeted systems [82, 83], tumor‐penetrating peptides, such as internalizing RGD (iRGD), to enhance deep tissue access [84], and stimuli‐responsive NPs capable of size or charge switching in acidic or protease‐rich TME [85]. iRGD contains the RGD motif that binds to α_v_ integrins and a C‐terminal CendR motif to enhance tissue penetration and internalization. Together, these approaches broaden the toolbox for overcoming physical barriers and improving CAR‐T cell infiltration into solid tumors.

There are other means to target the ECM. For example, researchers first administered a hydrogel intratumorally, which was loaded with l‐arginine and gold prodrugs (AuCl_4_^–^) [86]. This was followed by in situ synthesis of gold NPs throughout the tumor. Finally, microwave irradiation was applied, activating the gold NPs and enhancing the therapeutic effect through uniform heating and matrix remodeling. This system addressed ECM viscosity as a physical barrier to immune response by reducing components such as hyaluronic acid and collagen I, and by downregulating cell adhesion molecules. This remodeling process facilitated immune cell infiltration, such as CAR T cells, dendritic cells, M1 macrophages, and NK cells, effectively converting poorly immunogenic (“cold”) tumors into highly inflamed (“hot”) ones. The combination of microwave‐driven ECM modulation and CAR‐T cell therapy significantly improved antitumor efficacy, demonstrating a promising approach for clinical translation. Notably, microwave ablation has been successfully applied in clinical trials to treat deep‐seated tumors in organs, such as the liver, lung, and kidneys [87, 88].

Other approaches involve nanocarriers designed to target the disorganized vasculature of solid tumors by delivering agents that increase adhesion molecule expression or promote vascular normalization, thereby facilitating CAR‐T cell infiltration. For instance, one study exploited the overexpression of matrix metalloprotease 2 (MMP2) in the vascular endothelium and stroma of tumors [89]. Polymer NPs were loaded with an antiplatelet antibody and doxorubicin, and surrounded by a shell composed of MMP2‐cleavable peptides, lecithins, PEG‐functionalized phospholipids, and cholesterol. The NPs selectively depleted tumor‐associated platelets through MMP2‐triggered release of the antiplatelet antibody, leading to the disruption of the tumor vasculature and enhanced perfusion, and the delivery of doxorubicin, resulting in antitumor efficacy without systemic bleeding.

**FIGURE 2:**
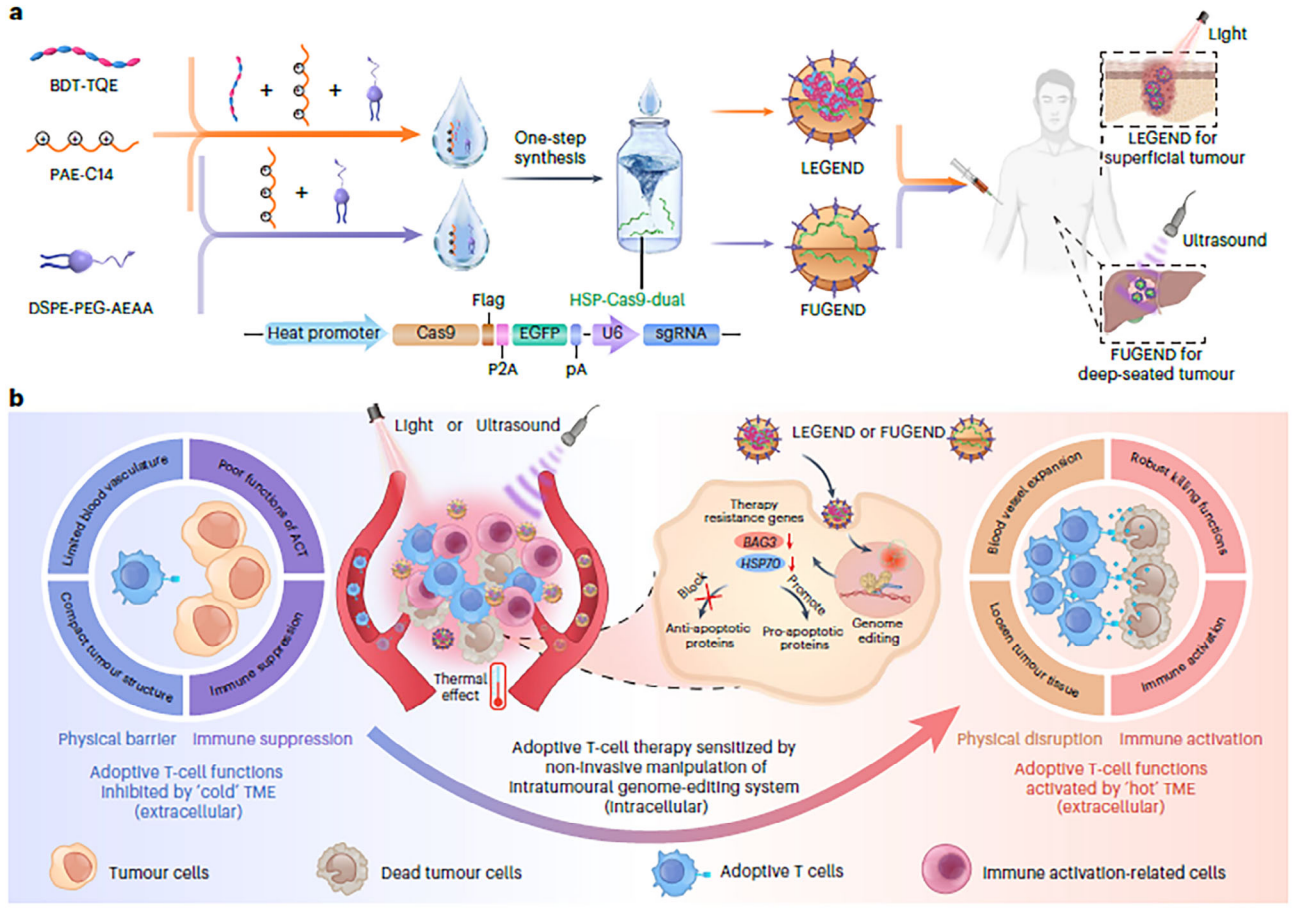
Development of a nanodevice to enhance the functions of adoptive T cells in solid tumors. (a) Illustration of the preparation of the nanodevice by one‐step coassembly of poly(β‐amino ester) (PAE‐C14), a semiconducting polymer (BDT‐TQE), a tumor‐targeting PEG‐functionalized phospholipid (DSPE‐PEG‐AEAA), and a heat‐inducible Cas9 plasmid encoding single guide RNA (sgRNA) of Hsp70 and BAG3 (HSP‐Cas9‐dual). LEGEND stands for light‐enabled genome‐editing nanodevice, and FUGEND for focused ultrasound‐enabled genome‐editing nanodevice. The preparation of FUGEND was similar to LEGEND apart from the absence of BDT‐TQE; thereby, PAE‐C14/DSPE‐PEG‐AEAA was used to encapsulate HSP‐Cas9‐dual. (b) Illustration of the intracellular genome editing of tumor cells and TME modulation mediated by the LEGEND or FUGEND nanodevices to promote the efficacy of adoptive T cells. Reproduced with permission [90]. Copyright 2023, Springer Nature.

Similarly, a system was developed exploiting previous research showing that Hsp70, a heat shock protein (HSP) that helps protect cells from stress, and BAG3 (a co‐chaperone protein involved in cellular stress responses and apoptosis regulation) are highly expressed in various types of cancer [90]. Their intracellular activity is crucial for cancer cell survival, and they modulate the TME to promote cancer progression and resistance to therapies [91, 92, 93]. Based on these findings, the authors developed a nanodevice for the delivery of a heat‐inducible CRISPR‐Cas9 system targeting Hsp70 and BAG3, which can be activated in vivo by a mild thermal effect induced by non‐invasive near‐infrared (NIR) light or focused ultrasound (Figure 2). After administration and intracellular delivery, the mild thermal stimulus not only triggered dual genome editing to disrupt the apoptotic resistance machinery of the tumor cells, but also remodeled the extracellular TME by breaking down physical barriers, such as dense ECM components that impede immune cell infiltration, and overcoming immunosuppression by reducing inhibitory signals within the TME, thereby creating a more permissive environment for ACT. Other strategies relying on heat generated by PTT or MH to remodel the TME are discussed in Section 3.

Beyond strategies to overcome the physical barriers of the TME, an elegant approach to modulate tumor acidity and hypoxia through chemical means using CaCO_3_ NP‐assembled colloidosomes encapsulating catalase has been proposed to jointly counteract tumor‐induced immunosuppression, thereby greatly enhancing the effectiveness of CAR‐T cell therapies against solid tumors [94]. Upon intratumoral administration, the colloidosomes could scavenge protons and decompose H_2_O_2_, owing to the properties of CaCO_3_ and catalase, respectively; resulting in simultaneous tumor acidity neutralization and tumor hypoxia attenuation. Alleviating tumor hypoxia subsequently reduced lactate production by altering intratumoral glucose metabolism, thereby reversing tumor immunosuppression. The treatment with the colloidosomes effectively stimulated both adaptive and innate antitumor immunity, characterized by (i) an increased CD3^+^CD8^+^ T cell‐to‐Treg ratio, (ii) enhanced NK cell infiltration, (iii) M2‐to‐M1 macrophage polarization, and (iv) elevated secretion of proinflammatory cytokines within the tumor. This treatment also substantially enhanced the therapeutic efficacy of epidermal growth factor receptor‐expressing CAR T cells against a human triple‐negative breast cancer xenograft by facilitating their tumor infiltration and promoting effector cytokine secretion, leading to tumor growth suppression.

### 2.2 Nanoparticles Delivering Immunomodulators to Enhance Adoptive Cell Therapy

**FIGURE 3:**
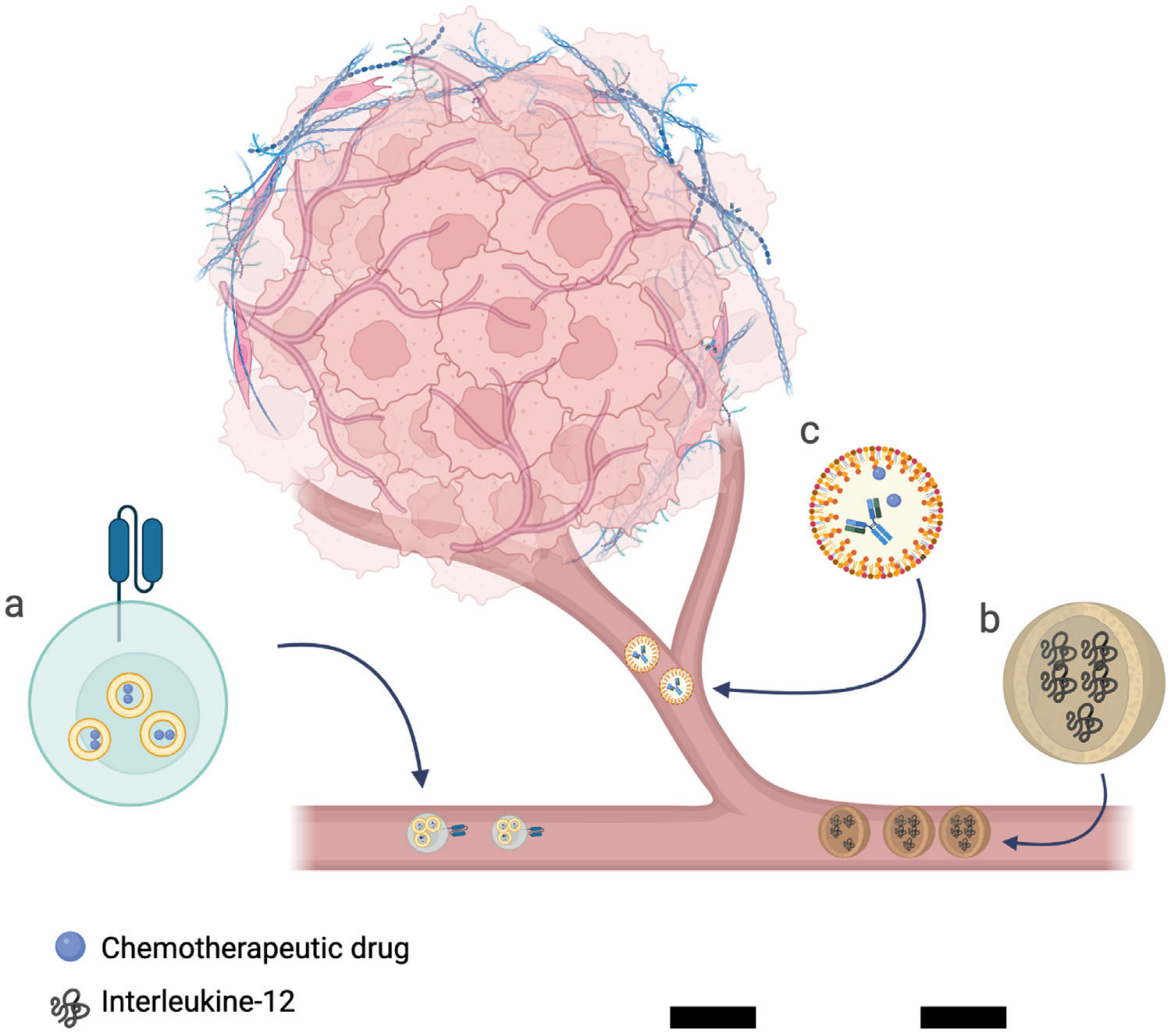
Schematic illustration of NP‐assisted strategies to enhance ACT efficacy in solid tumors. (a) “Cell hitchhiking” using immune cells (e.g., neutrophils, T cells) to deliver chemotherapeutic cargo. (b) NPs used as carriers for the delivery of immunomodulatory therapeutics (e.g., cytokines, TGF‐β inhibitors, TLR agonists, STING agonists). (c) NPs loaded with a chemotherapeutic drug and an antiplatelet antibody to enhance the delivery of the drug through disruption of the tumor vasculature and improved perfusion.

To overcome the multiple obstacles of the TME, the use of NPs for targeted delivery of immunomodulatory agents has gained significant attention thanks to several advantages they can offer over conventional systems, such as antibodies [95] and viral vectors [96]. Specifically, NPs can carry multiple therapeutic agents at the same time, potentially protecting them from degradation by the organism. Moreover, their “cargo” can be released in a site‐specific and stimuli‐controlled way, allowing the reduction of systemic toxicity [97] and improving therapeutic efficacy by concentrating immunomodulators within the TME [98]. In this context, several studies have demonstrated the versatility of NPs in addressing specific challenges in ACT, in particular, delivering immunomodulators to enhance ACT. This section details approaches using NP carriers for the delivery of different types of immunomodulators, including TGF‐β inhibitors, cytokines, TLR agonists, and STING agonists (Figure 3).

#### 2.2.1 TGF‐β Inhibitors

NPs designed to deliver TGF‐β pathway inhibitors can specifically target tumor‐associated fibroblasts, thereby reducing stromal fibrosis and creating a TME more permissive to T cell infiltration. For instance, researchers developed core‐shell NPs with a pH‐/H_2_O_2_‐responsive bovine serum albumin‐MoS_2_ shell encapsulating a low molecular polyethyleneimine complexed to siRNA targeting TGF‐β, aimed at remodeling the dense, immunosuppressive TME of triple‐negative breast cancer [99]. In this study, bovine serum albumin was exploited for albumin‐mediated tumor‐targeted delivery. Upon internalization in the lysosomes of cancer cells, in response to acid and H_2_O_2_, the degradation of MnO_2_ induced the generation of oxygen, which alleviated hypoxia, resulting in vasculature normalization and immunosuppression reversion. Hypoxia, which is a hallmark of most solid tumors due to rapid proliferation, upregulates hypoxia‐inducible factor 1α (HIF‐1α) and vascular endothelial growth factor, leading to abnormal vasculature, reduced vascular adhesion, recruitment of myeloid‐derived suppressor cells, and M2 polarization of tumor‐associated macrophages. Consequently, alleviating hypoxia is a strategy to create a more favorable microenvironment for CAR‐T cell therapy. Besides, the degradation of the MnO_2_ shell of the NPs also led to the delivery of TGF‐β siRNA to silence TGF‐β expression. Overall, this dual mechanism promoted vascular normalization and improved the penetration of both NPs and mesothelin‐targeted CAR T cells, leading to enhanced infiltration and antitumor efficacy in triple‐negative breast cancer models. Mesothelin is a relevant tumor‐associated antigen; it is a cell‐surface glycoprotein that is highly expressed in multiple cancers, including lung, pancreatic, and ovarian cancer. Importantly, the treatment with NPs and mesothelin‐targeted CAR T cells caused no significant body weight loss, no detectable histological damage to major organs, and no abnormalities in hepatic or renal function markers, indicating good biocompatibility and minimal systemic toxicity.

In a complementary approach, PEGylated liposomes encapsulating a TGF‐β inhibitor were designed to target melanoma‐specific CD8^+^ T cells via two strategies, using CD90, an internalizing receptor, or CD45, a non‐internalizing receptor [100]. This study allowed us to determine whether more sustained TGF‐β inhibition could be achieved either through liposomes bound to the surface of T cells, which were injected in mice and continuously released the drug for several days, or alternatively through liposomes internalized in the T cells and subsequently degraded via the endolysosomal pathway. Importantly, this work also compared the approach based on preloading the inhibitor‐loaded liposomes directly onto or into adoptively transferred T cells versus systemic injection of the liposomes to target T cells in vivo. This comparison highlighted that preloading ensured that every transferred T cell carries the therapeutic cargo, thereby enhancing consistency and potency, while the efficacy of systemic liposome administration was critically dictated by their biodistribution, given that targeting efficiency was intrinsically dependent on their in vivo biodistribution profile. Both approaches significantly improved T cell functionality, but the study underscored that targeting receptors exclusively expressed by the donor T cells and the mode of administration are critical for maximizing ACT efficacy, and that repeated administration of PEGylated liposomes could cause the development of anti‐liposome antibody responses.

In another study, TGF‐β blocking was combined with PTT to enhance tumor‐specific migration and long‐term antitumor efficacy of anti‐CD19 CAR T cells. Researchers designed amphipathic hydroxyethyl starch‐polycaprolactone NPs loaded with a TGF‐β receptor inhibitor (LY) and indocyanine green (ICG) [101]. The NPs were able to target lymphoma sites through the EPR effect. In NOD severe combined immunodeficient (SCID) gamma (NSG) Raji (human B lymphoma) tumor‐bearing mice, the LY/ICG‐loaded NPs showed enhanced and sustained tumor accumulation, in contrast to free ICG that was rapidly cleared from circulation. Upon NIR light irradiation, the release of the TGF‐β receptor inhibitor was enhanced owing to the local mild heat generated by ICG, inducing tumor cell destruction and disruption of the ECM, thus reducing the density of the compact stromal barrier and expanding tumor vasculature. Inhibiting TGF‐β also contributed to partial normalization of the tumor vasculature. As a result, this synergistic strategy combining photothermal and immunomodulatory effects allowed enhancing the therapeutic efficacy of CD19‐targeted CAR T cells towards lymphoma.

#### 2.2.2 Cytokines

An alternative way to overcome the immunosuppressive TME in solid tumors is the delivery of cytokines, such as interleukins and interferons, directly to the tumor site to increase immune cell attraction, activation, and infiltration, while minimizing systemic toxicity.

Cytokines are immunoregulatory proteins that mediate intercellular communication, including members of the interferon, interleukin, and tumor necrosis factor families [102]. In the TME, cytokine production is often impaired [103]. To address this challenge and meet clinical needs, several studies have described engineered cytokine‐based therapies designed to overcome rapid clearance and systemic toxicity, while enhancing synergy with immunotherapies, such as CAR T cells and ICIs [104, 105]. This concept has recently been applied in a clinical setting with CAR T cells engineered to secrete IL‐18, demonstrating improved efficacy in lymphoma patients refractory to previous CAR‐T cell therapy [106].

For example, IL‐12 is a potent cytokine that stimulates the production of IFN‐γ by T and NK cells. However, its clinical application has been limited by systemic toxicity, including on‐target, off‐tumor effects and a short half‐life, which reflect widespread immune activation rather than localized antitumor activity. To improve the delivery and therapeutic profile of cytokines, multiple groups have used nanocarriers, including liposomes, polymer NPs, and carbon nanotubes [103, 105, 107, 108].

Systemically administered immunomodulators, such as interleukins, are often limited by severe side effects. To address this, a redox‐responsive nanogel composed of cross‐linked human IL‐15 superagonist was developed. Thanks to the incorporation of a small proportion of anti‐CD45 in the nanogel, it could bind to the surface of endothelial growth factor receptor (EGFR)‐specific CAR T cells [109]. IL‐15 superagonists are engineered cytokine therapeutics that mimic and enhance the biological functions of native IL‐15, promoting robust activation and expansion of NK cells and CD8^+^ T cells. The nanogel was engineered using disulfide crosslinkers that can be cleaved in response to reducing conditions present on T cell surface induced by TCR activation. The cleavage of the disulfide bonds triggered the release of IL‐15 superagonist, enabling cytokine delivery to occur exclusively at the tumor site. Compared to systemic administration of free IL‐15, this strategy yielded 16‐fold greater expansion of T cells in the tumor of a human glioblastoma model in NSG mice and enabled the administration of cytokine doses up to 8 times higher. Notably, IL‐15 superagonist‐loaded nanogel remained stably associated with CAR T cells even during apoptosis, preventing premature cytokine release and systemic exposure. Whereas free IL‐15 superagonist induced body weight loss, systemic cytokine release, and elevated liver enzymes, the CAR T cell‐bound nanogel maintained basal serum cytokine levels, showed no overt toxicity, and permitted repeated dosing, advancing safe and selective immune stimulation while delaying tumor growth.

Another method for the delivery of cytokines involved a redox‐responsive human serum albumin NPs loaded with IL‐12 that were conjugated to CAR T cells without influencing their antitumoral activity [110]. The NPs released IL‐12 selectively in the presence of the increased thiol groups on the cell surface, with a redox mechanism similar to the one described in the previous paragraph. The release of IL‐12 enabled the production of chemokines (C−C chemokine ligand 5 (CCL5), CCL2, CXCL10) and further increased the presence of CD8^+^ CAR T cells. In Raji tumor‐bearing immunodeficient mice, this strategy led to a 10‐fold increase in CD8^+^ CAR‐T cell accumulation in the tumor, in an increased proliferation and production of antitumor cytokines, resulting in tumor growth suppression. It is noteworthy that this NP‐based “backpacking” (or alternatively named hitchhiking) approach improved biosafety compared with free IL‐12 administration, as systemic levels of proinflammatory cytokines (TNF‐α, IFN‐γ, IL‐2, IL‐1β, and IL‐6) remained at baseline. The toxicity was mitigated, with alanine aminotransferase and aspartate aminotransferase levels comparable to controls (i.e., phosphate‐buffered saline (PBS), CAR T cells only), and no histopathological liver damage was observed. This cell approach based on the loading of NPs in cells is highly promising, but it is limited by dilution of the backpacked NPs due to stimulation of cell division. In addition, the potential effects of NPs on T cell proliferation, differentiation, metabolism, and exhaustion should be investigated.

While these strategies enhance intratumoral cytokine exposure and reduce systemic inflammation in preclinical models, they also introduce important translational challenges. NP loading may dilute with T cell proliferation, limit durability of cytokine delivery, or interfere with T cell fitness and trafficking.

#### 2.2.3 TLR Agonists

TLR agonists have been explored in clinical trials and have shown efficacy in reprogramming the TME. These agents promote a pro‐inflammatory milieu at the tumor site, enhance infiltration of effector T cells, and induce the production of type I interferons [111, 112]. For instance, combined TLR stimulation with copper diethyldithiocarbamate‐loaded liposomes delivered via the bacterium M. gryphiswaldense was reported. Bacteria possess a wide range of pathogen‐associated molecular patterns that are recognized by TLRs on immune cells, triggering an inflammatory response. Using the B16F10 melanoma model, a tumor known for developing resistance to ICIs, a pronounced antitumor effect was obtained, which exceeded the typical impact of bacterial TLR activation alone [113]. Although TLRs are predominantly expressed on innate immune cells, studies have shown that TLR signaling can also directly contribute to T cell costimulation, enhancing T cell survival, proliferation, cytokine production, and the transition to memory phenotypes. This approach holds promise as an adjuvant or preconditioning regimen in combination with ACT, particularly in CAR‐T cell‐based treatments.

A novel synergistic targeted antitumor immunotherapy approach was developed by combining a tumor‐specific antigen‐based nano‐adjuvanted vaccine with anti‐programmed cell death protein 1 (PD‐1) antibody and CD16 CAR‐T cell therapy [114]. CD16, a transmembrane Fc receptor expressed on large granular lymphocytes, is known to play a key role in antibody‐dependent cellular cytotoxicity. In this study, NPs made of the self‐assembly of protamine sulfate and carboxymethyl β‐glucan were loaded with a CpG oligodeoxynucleotides and mixed with mouse melanoma‐derived antigens of tumor cell lysates and neoantigens. Oligodeoxynucleotides with unmethylated CpG motifs have been identified as TLR9 agonists capable of activating both innate and adaptive immune responses. The NPs were able not only to target the delivery of CpG oligodeoxynucleotides into dendritic cells, markedly inducing their activation and maturation, but also to enhance the antitumor efficacy of an anti‐PD‐1 antibody by remodeling the TME and promoting substantial infiltration of CD3^+^CD8^+^ T cells into tumor tissues. Moreover, the humoral immune responses induced by the cytotoxic activity of T cells specifically engaged CD16 CAR T cells, markedly inhibiting tumor growth in melanoma‐bearing mice through an antibody‐dependent cellular cytotoxicity pathway. The NPs also exhibited a favorable safety profile: after subcutaneous injection, strong fluorescence was detected in draining lymph nodes at 24 and 72 h, indicating efficient and sustained delivery of CpG oligodeoxynucleotides without off‐target accumulation, and no overt signs of systemic toxicity were observed in treated mice. Moreover, the enhanced antitumor efficacy of CpG‐loaded NPs + anti‐PD‐1 antibody treatment was accompanied by significantly increased IFN‐γ levels (approximately 2.5‐fold higher than free CpG + anti‐PD‐1) and improved survival rates, demonstrating that the NPs were both effective and well‐tolerated in vivo. This elegant approach demonstrated that a vaccine inducing antimelanoma antibodies can be simultaneously used with CAR T cells in synergistic targeted antitumor immunotherapy.

While TLR agonists can potently stimulate antitumor immunity, excessive or systemic activation may trigger T cell apoptosis, cytokine‐induced toxicity, or rebound immunosuppression, limiting therapeutic efficacy. Overstimulation can also induce regulatory pathways or myeloid‐derived suppressor cell expansion, which counteract the intended immune response. NP‐based delivery of TLR agonists offers a potential solution by localizing TLR agonist release, controlling dosing kinetics, and targeting immune cells within the TME.

#### 2.2.4 STING Agonists

Another promising immunomodulatory pathway involves the cyclic GMP‐AMP synthase (cGAS)‐STING axis. STING activation leads to type I interferon and chemokine production, facilitating the recruitment of innate and adaptive immune cells while suppressing immunosuppressive populations, such as Tregs and myeloid‐derived suppressor cells [115]. However, clinical translation of STING agonists faces limitations, including poor bioavailability, short half‐life, off‐target toxicity, and upregulation of programmed cell death ligand 1 (PD‐L1), which contributes to T cell exhaustion [116]. To overcome this hurdle, nanocarriers hold significant promise, given their high drug‐loading capacity, tunable surface properties, and ability to improve drug pharmacokinetics and tumor targeting [117]. Recent work has reviewed a range of nanocarriers engineered for efficient delivery of STING agonists [115].

A relevant example is the encapsulation of a non‐nucleotide STING agonist, MSA‐2, into liposomes, generating SAProsomes (STING‐Activating Pro‐drug liposomal vesicles) [118]. In a mouse tumor model bearing Lewis lung carcinoma, a durable tumor regression was achieved in 67% of treated mice without weight loss. A combination therapy with anti‐PD‐L1 antibodies and SAProsome‐3 led to a high tumor growth inhibition, compared to only 24% when using free MSA‐2 and anti‐PD‐L1. This result was associated with increased immune cell infiltration, M1 macrophage polarization, and a transition from cold to hot tumor phenotypes. These findings highlight the importance of SAProsomes as a powerful delivery platform for STING agonists to stimulate the secretion of inflammatory cytokines. By improving drug stability, tumor targeting, and immune activation, SAProsomes significantly enhanced therapeutic outcomes, transforming cold tumors into hot tumors and boosting the efficacy of ICIs such as anti‐PD‐L1. This approach represents a promising combinatory approach for ICIs, and it could be applied with ACT, such as CAR‐T cell therapy.

The combination of CAR‐T cell therapy with the STING agonist IMSA101, a 2’3’‐cyclic guanosine monophosphate‐adenosine monophosphate (cGAMP) analog, administered intratumorally, was reported to improve tumor control and extend survival in mouse models [119]. The STING activation boosted CAR‐T cell activity by inducing IL‐18 production, which enhanced T cell activation and infiltration. Interestingly, using CAR T cells with IL‐18 receptor signaling knockout reduced the effect, but did not completely abolish it, highlighting the contributory role of IL‐18 in the observed synergy as well as other independent mechanisms. These results support the potential of STING agonists to enhance CAR‐T cell therapy for solid tumors.

To further address the immune suppression of T cells, strategies that rely not only on ICIs, but also aim at locally reversing immunosuppressive mechanisms have been developed against solid tumors. For instance, one approach involved the use of inhalable cell membrane‐derived nanovesicles prepared from T cells that express anti‐PD‐L1 single‐chain variable fragments (scFv) [120]. The nanovesicles transporting an anti‐PD‐L1 scFv were loaded with a STING agonist (cGAMP). STING agonists are known to induce antitumor immunity by activating dendritic cells, promoting type I interferon production, and enhancing T cell recruitment and activation. Notably, in the TME, dendritic cells frequently overexpress PD‐L1, making them primary targets for both the anti‐PD‐L1 scFv and cGAMP‐mediated STING activation. In this study, the nanovesicles were administered via inhalation and selectively accumulated in the lungs, 5.5‐fold higher compared to intravenous delivery, minimizing systemic toxicity and releasing cGAMP to tumor cells that overexpress PD‐L1. The activation of the STING pathway induced strong interferon responses, 2‐2.9‐fold higher than free cGAMP, despite a 50‐fold lower dose, and reduced immunosuppressive cells within the TME. The remodeling of the TME (increasing pro‐inflammatory Th1 and Th17 responses, suppressing Th2 polarization, and promoting M1 macrophage infiltration) enhanced mesothelin‐targeted CAR‐T cell infiltration (administered 24 h after nanovesicle inhalation). The PD‐L1 blocking epitope further contributed to checkpoint inhibition directly within the tumor site. The treatment showed an improved survival rate in a lung metastasis mouse model without tumor recurrence compared to the mice treated with CAR T cells only over 50 days. The nanovesicles exhibited high specificity for PD‐L1‐expressing cells, maintained stable size and dispersion with negligible cGAMP leakage over time, indicating a favorable safety and stability profile for clinical applications.

Overall, STING agonists represent a promising adjuvant approach for enhancing cancer immunotherapy, including CAR‐T cell therapy. Nanocarriers can significantly improve the specificity and efficacy of STING agonists. Nevertheless, despite strong preclinical efficacy, the clinical translation of STING agonists remains challenging. STING signaling is highly context‐ and dose‐dependent, and excessive activation can induce systemic inflammation, immune exhaustion, and tissue toxicity, which are concerns that are amplified when combined with ACT. In addition, pronounced species‐specific differences in STING pathway activation limit the predictive value of murine models and may partly explain the modest outcomes observed in early clinical trials.

#### 2.2.5 Other Immunomodulation Strategies

To address the challenge of the immunosuppressive TME, pH‐responsive NPs made of the co‐assembly of Fmoc‐histidine, Mn^2+^, CCL5, and NLG919 (an inhibitor of the indoleamine‐2,3‐dioxygenase pathway), were designed to release CCL5 and NLG919 in the tumor owing to the slight acidity of the TME that induced NP disassembly. A reduction in C–C chemokine ligands, such as CCL5, within tumor tissue is known to lead to the depletion of tumor‐infiltrating lymphocytes [66], while indoleamine 2,3‐dioxygenase suppresses T cell activation. Therefore, the aim of co‐delivering CCL5 and NLG919 was to enhance chemotaxis and reverse indoleamine‐2,3‐dioxygenase‐mediated immunosuppression, respectively, thus promoting a TME favorable to optimal CAR‐T cell‐mediated cytotoxicity. Indeed, the administration of the immunomodulatory NPs intraperitoneally in a lung animal cancer model, followed by intraperitoneal injection of CAR T cells targeting CD276, which is overexpressed in many solid tumors, resulted in a significant improvement of CAR‐T cell infiltration and tumor growth inhibition.

An alternative approach to overcome the immunosuppressive TME in solid tumors involves the targeting of immune checkpoints [121]. Nevertheless, systemic administration of ICIs alters immune homeostasis and causes autoimmune responses. As an example, magnetic Fe_3_O_4_ nanoclusters were functionalized with an anti‐PD‐1 antibody through a pH‐responsive imine bond [122]. After binding of the NPs to effector T cells via PD‐1 recognition in vitro, the cells were administered intravenously in an E.G7‐OVA subcutaneous lymphoma‐bearing mouse model using immunocompetent mice, and, upon the application of an external magnetic field, they were targeted to the tumor site under MRI guidance. Quantitative biodistribution analyses using in vivo fluorescence imaging, ex vivo organ imaging, and inductively coupled plasma mass spectrometry revealed a threefold increase in T cell accumulation in tumors under magnetic guidance, accompanied by reduced off‐target distribution in major organs such as the liver, spleen, lung, and kidneys. The acidic conditions of the TME caused the hydrolysis of the imine bond, releasing the anti‐PD‐1 antibody for PD‐1 blocking, thereby enabling localized checkpoint inhibition. The simultaneous presence of T cells and checkpoint inhibitors led to enhanced cytotoxicity of T cells in vitro and suppression of the tumor development in vivo. Importantly, short‐term safety evaluation demonstrated minimal acute toxicity, as evidenced by stable body weight and temperature, absence of cytokine storm indicators (IL‐6 and TNF‐α), normal blood biochemical parameters, and no observable histopathological damage in major organs following treatment. This targeted strategy allowed for the possibility of reducing the administered dose of T cells and anti‐PD‐1 antibodies. This approach was also extended to adoptive tumor‐infiltrating lymphocyte therapy. Similarly, tumor‐infiltrating lymphocytes extracted from 4T1 breast tumor tissues were bound to the anti‐PD‐1 antibody‐magnetic nanoclusters and administered intravenously to the same tumor mouse model, and a magnetic field was applied for tumor accumulation. An almost complete tumor growth inhibition was observed, along with a very high mouse survival and no signs of metastasis, whereas the administration of tumor‐infiltrating lymphocytes alone led to little inhibition. Short‐term safety evaluations demonstrated stable body weight and temperature, no elevation of systemic inflammatory cytokines (IL‐6 and TNF‐α), normal blood biochemical parameters, and no detectable histopathological damage in major organs, indicating minimal acute toxicity of the nanocluster‐based system. This targeted strategy also enabled dose reduction of both T cells and anti‐PD‐1 antibodies. This result, although it was obtained in controlled, homogeneous immunodeficient mouse models, confirmed the higher efficiency of this approach over traditional ACT. Nonetheless, potential hazard evaluation associated with this system would be necessary, considering the risk of oxidative stress induction [123], bioaccumulation, and abnormal biodistribution associated with Fe_3_O_4_ NPs [124].

In addition to inorganic NPs, LNPs can also be used as carriers for delivering T cells and agents capable of modulating the immunosuppressive TME in solid tumors. For instance, a PD‐1 checkpoint‐targeted strategy to enhance ACT was designed by encapsulating LNPs with an inhibitor of SHP2, a protein tyrosine phosphatase involved in key cellular processes that govern cell survival and proliferation [125]. SHP2 plays a central role in connecting multiple signaling pathways triggered by growth factors, cytokines, and ECM receptors, including the JAK/STAT, PI3K/AKT, and PD‐1/PD‐L1 pathways [126]. When PD‐1 binds to its ligand, it forms negative costimulatory microclusters that suppress T cell receptor signaling through the recruitment of SHP2. Similarly, other immune checkpoints, including CTLA‐4 and BTLA, also recruit SHP2 within their cytoplasmic domains to inhibit T cell activity. In this study, the NPs loaded with the SHP2 inhibitor were encapsulated in T cells and administered intravenously in an E.G7‐OVA subcutaneous tumor mouse model, allowing for improved cytolytic activity of T cells through PD‐1/PD‐L1 checkpoint inhibition, demonstrating tumor eradication with a very high survival rate and without tumor recurrence. Short‐term biodistribution analyses showed that NP loading did not alter the physiological biodistribution and proliferation of T cells. Furthermore, acute safety and immunocompatibility assessments revealed preserved T cell viability and no significant body weight loss. Although promising, these outcomes should be intended more as preclinical rather than evidence of clinical performance, considering that they were obtained in a controlled and homogeneous murine model that does not often reflect the complexity of human solid tumors.

**FIGURE 4:**
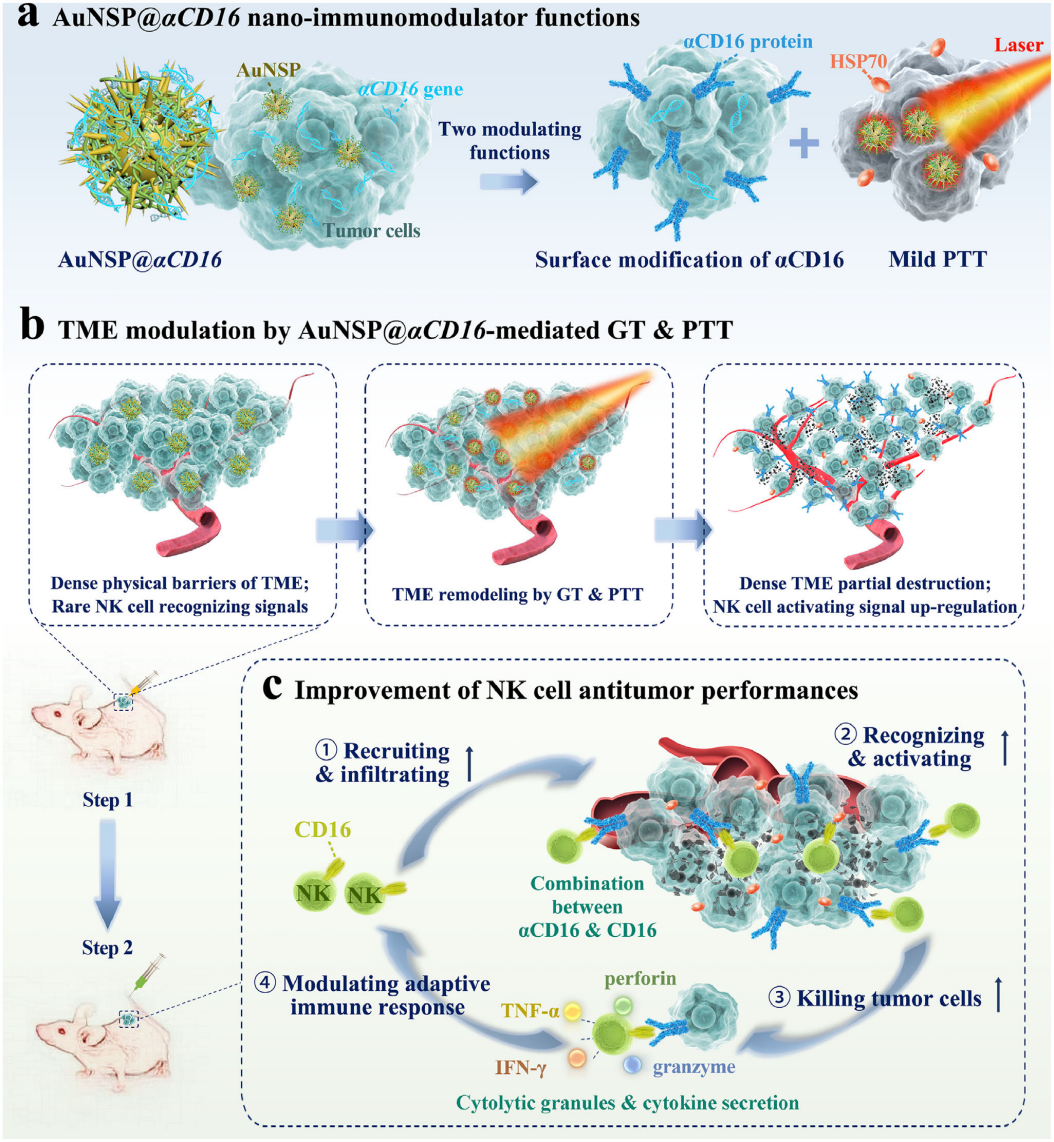
Illustration of gold NPs functionalized with an αCD16 encoding plasmid (AuNSP@αCD16)‐mediated modulation of the TME to increase antitumor activity of NK cells. (a) Role of AuNSP@αCD16 as a nano‐immunomodulator: surface modification of the tumor via αCD16 and induction of mild PTT under NIR light irradiation. (b) AuNSP@αCD16‐mediated modulation of the TME: disruption of the dense physical barrier and amplification of NK cell recognition and activation pathways. (c) Subsequent enhanced NK cell antitumor efficacy. GT stands for gene transfection. Reproduced with permission [127]. Copyright 2022, Elsevier.

Metallic NPs can also be exploited for their capacity to generate heat by MH and/or PTT. In this regard, gold NPs with a polycationic shell complexing an αCD16 encoding plasmid were exploited to enhance adoptive NK cell immunotherapy in a solid tumor through combined PTT and gene therapy (Figure 4) [127]. The therapeutic potential of NK cells faces major challenges due to the physical barrier of the TME and the absence of necessary recognition signals for NK cells on tumor cells. In this study, αCD16 gene transfection, mediated by the NPs, allowed modifying the surface of tumor cells with the CD16 antibody for NK cell recognition. Indeed, NK cell binding to tumor cells in vitro increased to 20% compared to 12% in the control group. NK cell activation through CD16‐mediated antibody‐dependent cellular cytotoxicity resulted in the release of cytolytic granules to promote the lysis of the tumor cells and secretion of proinflammatory cytokines to modulate adaptive immune responses. In addition, the gold NPs were used as a photothermal agent to disrupt the dense physical barrier by PTT, thereby creating a more permeable TME that facilitated NK cell infiltration. Tumor growth inhibition was observed in a HeLa tumor‐bearing mouse model after intratumoral administration of the NPs followed by intravenous injection of NK cells, while a remarkable growth delay was also obtained in the absence of laser irradiation, confirming that the modification of tumor cell surface with αCD16 antibody can enhance the antitumor effects of NK cells. Notably, gold NP‐mediated photothermal intervention caused no significant body weight loss in the mice, no obvious histopathological changes in major organs, and serum biochemistry indicators remained within normal ranges. Local intratumoral administration ensured high NP concentrations at the tumor site while limiting systemic exposure, indicating a favorable safety profile. This mode of administration limited the systemic toxicity often associated with intravenously delivered NPs, even though a fraction of locally injected NPs may enter systemic circulation, presenting a risk of off‐target effects in healthy tissues. An inherent limitation of this approach, based on local administration and PTT, is the poor applicability for deep tumors.

Liposomes have also been used to remodel the TME without causing systemic toxicities. One approach involved the development of iRGD‐targeted liposomes carrying both a phosphoinositide 3‐kinase (PI3K) inhibitor and 7DW8‐5, a NK T cell agonist [128]. PI3K inhibition selectively targets immunosuppressive populations, such as tumor‐associated macrophages and monocytic myeloid‐derived suppressor cells, decreasing immune suppression. The treatment enabled the removal of protumor cell populations and the stimulation of antitumor effector cells. It allowed a therapeutic window of two weeks during which tyrosine kinase‐like orphan receptor (ROR1)‐specific CAR T cells exhibited curative effects. ROR1 is strongly expressed on the surface of breast cancer cells. In a syngeneic breast cancer mouse model, tumor clearance was reached for 50% of the mice, while the other half showed a tumor regression, resulting in a doubling in survival, compared to conventional CAR‐T cell therapy. Safety studies confirmed that repeated injections of liposomes were well tolerated with no weight loss. Histopathologic evaluation revealed only minor mononuclear infiltrates in the liver and kidneys, occasional hepatocellular mitoses, and subtle kidney infiltrates with no necrosis or proteinuria. Blood analyses showed mild leukocytosis with neutrophilia, monocytosis, and lymphocytosis, and minimally elevated alanine transaminase levels, while glucose and other serum chemistry parameters remained normal. Empty liposomes used as controls exhibited no adverse effects, indicating that these nanocarriers are biocompatible and safe for repeated dosing.

Another biological delivery platform was developed to address the issue of an immunosuppressive TME using CD19‐specific CAR T cells functionalized with liposomes loaded with SCH‐58261, a specific antagonist of the A2 adenosine receptor, known to inhibit T cell activity [129]. The liposomes were engineered to include lipids functionalized with maleimide, which enabled a stable covalent attachment to the free thiol groups present on the surface of the CAR T cells, without altering their viability, migratory capacity, and cytotoxic function. The liposome‐bound CAR T cells achieved higher tumor accumulation with reduced liver uptake compared to free liposomes. They showed a significant tumor growth inhibition in a murine model of ovarian cancer. Nevertheless, the long‐term fate of the liposomes remains unexplored and should be addressed, considering that long‐term biodistribution and clearance depend on size, composition, and dose [130, 131].

Another emerging immunomodulatory approach involves NP‐mediated delivery of epigenetic modulators to prevent T cell exhaustion [132]. Exhausted T cells display stable epigenetic programs, including DNA hypermethylation at effector loci, which restricts their capacity to regain cytotoxic function [133]. Systemic administration of DNA methyltransferase inhibitors (DNMTi), such as decitabine or azacitidine, is limited by short half‐life and hematologic toxicity [134]. NP‐based delivery systems can enhance tumor‐specific biodistribution, protect DNMTi from rapid degradation, and enable controlled release within the TME. Recent studies using polymeric or LNPs encapsulating DNMTi have demonstrated restored effector gene expression, reduced exhaustion markers (PD‐1, TIM‐3, LAG‐3), and improved persistence and infiltration of CAR T cells [132, 135, 136].

Targeting metabolic competition in the TME represents another promising avenue to enhance ACT persistence. Tumor cells consume glucose and amino acids while releasing lactate, depriving T cells of essential substrates and promoting exhaustion [137]. NPs have been explored to deliver metabolic substrates to sustain glycolysis, encapsulate mitochondria‐targeted antioxidants to preserve oxidative phosphorylation [138], or inhibit lactate accumulation to restore effector function [139]. These approaches highlight NP‐assisted metabolic reprogramming as a promising complement to ACT persistence.

Beyond reshaping the immunosuppressive TME, NP‐based immunomodulation may also strengthen off‐the‐shelf CAR‐T cell therapy by reducing the host‐versus‐graft responses that eliminate allogeneic cells. CAR T cells require extensive gene editing, such as TCR targeting, β2‐microglobulin knockout, and HLA silencing, to prevent graft‐versus‐host disease and limit rejection [140]. Yet, allogeneic CAR T cells still persist for only about 28 days due to immune clearance and the continued need for intensive lymphodepletion [141]. Localized delivery of immunosuppressor‐loaded NPs offers a promising strategy to overcome the limitations of systemic administration by providing site‐specific immunosuppression with reduced systemic toxicity [142].

While NP‐enabled strategies show promise in overcoming physical barriers, some limitations must be acknowledged. Enzymatic ECM remodeling may risk off‐target tissue damage and variable efficacy across tumor types [143]. Vascular normalization approaches often face transient effects and heterogeneity in vessel response [144, 145]. Stromal depletion strategies targeting cancer‐associated fibroblasts raise concerns about systemic toxicity and loss of normal fibroblast functions [146]. Tumor‐penetrating peptides, such as iRGD, can enhance intratumoral access, but may also facilitate unwanted spread of therapeutic agents [147]. Stimuli‐responsive NPs, although innovative, require precise control of activation conditions and raise questions about reproducibility and safety in clinical settings [148]. Addressing these uncertainties and translational barriers is essential to ensure that promising preclinical findings can be safely and effectively translated into clinical practice.

### 2.3 Combining Adoptive Cell Therapy and Chemotherapeutic Drug Delivery

Combination strategies to overcome the various hurdles of the TME have been explored, particularly through the integration with other cancer therapies [20]. This section describes the combination of ACT and delivery of chemotherapy drugs using NPs as carriers (Figure 3). Various classes of chemotherapeutic agents, including alkylating agents (e.g., cyclophosphamide), platinum compounds (e.g., cisplatin, carboplatin, oxaliplatin), antimetabolites (e.g., methotrexate), anthracyclines, DNA methyltransferase inhibitors, and spindle poisons (e.g., taxanes), have long been employed in clinical oncology [149]. However, systemic chemotherapy may also exert cytotoxic effects on the transferred CAR T cells, both in circulation and at the tumor site. Moreover, it may negatively impact the endogenous T cell pool, particularly those generated in the bone marrow. As conventional chemotherapeutics do not exclusively target malignant cells, off‐target toxicities remain a significant concern [150]. To address these limitations, several innovative strategies encompassing the encapsulation of chemotherapeutic agents in nanocarriers have been developed to enhance tumor‐specific delivery while minimizing systemic exposure [71, 151]. The following sections will discuss some approaches for the combination of chemotherapy and cell therapies. While some of these strategies do not directly employ ACT, they offer valuable conceptual frameworks and lessons that may inform the design of future ACT‐based delivery approaches.

In some clinical trials, lymphodepleting chemotherapy administered prior to CAR‐T cell infusion has shown benefits by reducing endogenous immune cells, including immunosuppressive populations, such as Tregs and myeloid‐derived suppressor cells, thereby enhancing CAR‐T cell engraftment and expansion. This preparative regimen is commonly used in hematological malignancies and remains a key strategy to improve CAR‐T cell efficacy [152, 153, 154]. Among the commonly used conditioning regimens, the combination of cyclophosphamide and fludarabine has shown superior efficacy in promoting CAR‐T cell expansion, persistence, and infiltration into the TME. However, these lymphodepleting protocols exert broad, non‐specific suppression not only of lymphocytes but also of hematopoietic stem cells, contributing to significant toxicity [152]. To date, the only targeted lymphodepleting agent in clinical use is alemtuzumab, a monoclonal antibody against CD52 expressed on lymphocytes. This highlights the need for novel engineering approaches, for instance, using NPs as drug carriers, to increase specificity and reduce off‐target effects associated with current lymphodepletion approaches [155].

An innovative approach in the context of chemotherapy relies on the use of LNPs to deliver an mRNA encoding Escherichia coli purine nucleoside phosphorylase (PNP) in solid tumors via an intratumoral injection [156]. The PNP enzyme can convert fludarabine prodrug into the cytotoxic metabolite 2‐fluoroadenine. Since PNP is not present in human cells and fludarabine is a poor substrate for human PNP analogs, this strategy ensures selective activation at the tumor site, minimizing off‐target effects. This elegant approach could be combined with ACT for a superior anticancer effect.

Cell hitchhiking strategies have also been investigated, for example, PEGylated liposomes covalently conjugated onto the surface of the magnetotactic bacterium *M. gryphiswaldense* using triazine as a crosslinker [113]. The liposomes, loaded with an anticancer drug (copper diethyldithiocarbamate), leveraged bacterial motility to facilitate targeted drug delivery, resulting in significant tumor growth reduction, demonstrating potential as an anticancer strategy. The bacteria also induced a systemic immune response directed primarily against the bacteria, with collateral impact on the tumor. The presence of the liposomes on the surface of the bacteria allowed enhancing bacterial stealth properties thanks to the PEG chains on the liposomes, thus prolonging blood circulation time and improving accumulation in the tumor.

Not only bacteria, but also endogenous leukocytes can be exploited as carriers of a chemotherapeutic drug. As an example, to achieve both effective penetration of the blood‐brain barrier and delivery of a chemotherapeutic agent to glioblastoma tumors, paclitaxel encapsulated‐liposomes were internalized by neutrophils, generating neutrophil‐based delivery vehicles [157]. Leveraging the natural ability of neutrophils to penetrate inflamed brain tumors, as inflammatory factors released after tumor resection direct neutrophil migration in the inflamed brain, this platform enabled targeted delivery of paclitaxel for postoperative glioma treatment. Excessive activation of neutrophils by elevated inflammatory cytokines in the inflamed brain induced their disruption, resulting in the release of liposomal paclitaxel.

A cell hitchhiking strategy has also been reported using CAR T cells. In this case, a synergy between TQM‐13 (targeted quadruple mutant of IL‐13) CAR T cells and chemotherapy was achieved by conjugating fluorescent polymer‐polylactide copolymer NPs loaded with doxorubicin to CAR T cells via a pH‐sensitive hydrazone linker using an azide‐alkyne cycloaddition click reaction [158]. TQM‐13 CAR T cells have a high binding affinity to glioblastoma cells expressing IL13Rα2. When the NP‐modified CAR T cells were administered intravenously in a glioblastoma tumor mouse model, enhanced delivery of doxorubicin was observed into the brain tumor, surpassing the efficiency of NP injection alone. This strategy not only minimized systemic toxicity of the anticancer drug, but also enhanced CAR‐T cell penetration across the blood‐brain barrier by leveraging the natural ability of T cells to extravasate through the blood‐brain barrier [159].

While chemotherapeutic regimens remain a critical component of current CAR‐T cell therapy protocols, their optimization is crucial to minimize toxicity and preserve CAR‐T cell functionality. Emerging targeted drug delivery approaches using NPs offer promising avenues to synergize with ACT modalities, specifically with CAR T cells, while mitigating off‐target effects.

NP‐based strategies potentially represent a powerful and versatile methodology to overcome the challenges of ACT in solid tumors. Preclinical studies demonstrate that NPs can enable localized, stimulus‐responsive delivery of immunomodulators, ICIs, and cytokines, enhancing T cell infiltration, persistence, and cytotoxicity in the immunosuppressive TME, while minimizing systemic toxicity. From metallic NPs to lipid‐based and protein‐derived carriers, these platforms offer new ways to integrate innate and adaptive immune responses, resulting in improved tumor eradication and survival in preclinical models. However, clinical translation faces significant obstacles, including scalable manufacturing, batch‐to‐batch reproducibility, regulatory complexity, potential immunogenicity, and long‐term toxicity and off‐target accumulation. Among available NPs, LNPs delivering cytokines or ICIs appear most clinically feasible due to established production protocols, biocompatibility, and favorable pharmacokinetics. Addressing these challenges will require standardized good manufacturing practice production, careful NP selection to minimize immunogenicity, thorough evaluation of nano‐biointeractions, and rigorous monitoring of biodistribution and immune responses in early‐phase trials. Systematic optimization of these parameters is essential to realize the translational potential of NP‐mediated ACT strategies.

After addressing how NPs enhance ACT through drug delivery, the next section examines strategies to further improve ACT by leveraging the intrinsic properties of NPs for hyperthermia.

## 3 Combination of Adoptive Cell Therapy and Nanoparticle‐Mediated Hyperthermia

**FIGURE 5:**
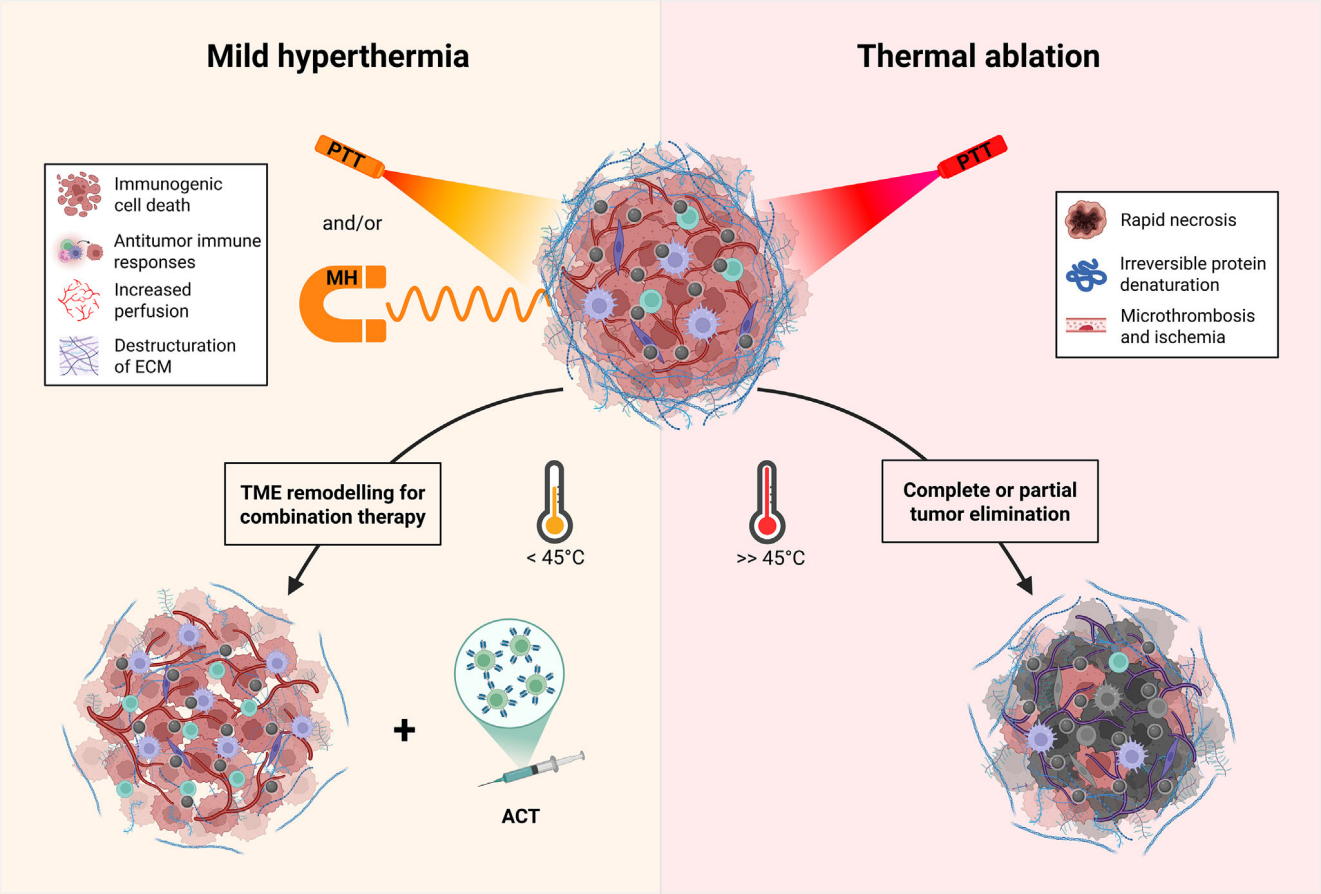
Effect of PTT and/or MH on the TME depending on the temperature reached in the tumor (mild hyperthermia vs. thermal ablation).

This section describes strategies to enhance ACT by hyperthermia, namely PTT and MH mediated by NPs (Figure 5). The effects of the local temperature increase on both the physical and immunosuppressive barrier of the TME are also discussed, along with the impact on tumor growth.

### 3.1 Combination of Adoptive Cell Therapy and Photothermal Therapy

Hyperthermia therapy is a hot topic within oncology, exploiting elevated temperatures to modulate or eradicate tumor tissue [160]. NP‐mediated PTT is a localized hyperthermia strategy using light‐absorbing photothermal agents as a heating source [161]. By concentrating photothermal agents in malignant tumor tissue, temperature can be locally increased by efficient heat generation and dissipation upon laser irradiation, with minimal thermal damage to surrounding, healthy tissue [162, 163]. The most commonly applied excitation source for PTT is NIR light, showing superior tissue‐penetration properties compared to UV and visible light, while exhibiting minimal photodamage to overlying and surrounding tissue [164, 165, 166]. Nevertheless, NIR light penetrates only a few cm through tissue, depending on the tissue composition and NIR wavelength [167]. Consequently, PTT treatment is limited to superficial tumors or tumors that can be accessed by endoscopy or interstitial optical fibers [168]. In contrast, deep‐seated or diffuse tumors remain difficult to treat with PTT. Photothermal agents encompass organic molecules, such as ICG [101, 169, 170]. and inorganic nanomaterials [171], including carbon nanotubes [172, 173, 174], gold NPs [175, 176]. and metal oxide NPs [177, 178, 179]. By monitoring the temperature of the tumor during exposure, the laser irradiation power density can be modulated in real time to maintain thermal exposure within a defined therapeutic temperature range. However, the extent of thermal damage is not only dependent on the target temperature, but also on the thermal dose, which reflects the full temperature profile over the total exposure time. A commonly used metric in clinical practice to quantify the thermal dose during hyperthermia treatment is the cumulative equivalent minutes at 43°C (CEM43°C), in which the measured tumor temperature is integrated over time and converted to the equivalent time of heating at 43°C [180, 181, 182]. Despite CEM43°C presenting a practical proxy to quantify and compare the thermal doses applied across different photothermal agents and laser exposure parameters, there are few PTT studies using CEM43°C to evaluate and quantify their results, making it challenging to compare findings in which different temperature‐time profiles are applied. In most cases, PTT‐induced heating is reported using temporal temperature plots, along with exposure duration and target temperature, which are thus the proxies that will be primarily referred to in this section. Based on these parameters, there are mainly two distinctly defined strategies for PTT, namely thermal ablation and mild hyperthermia (Figure 5).

**FIGURE 6:**
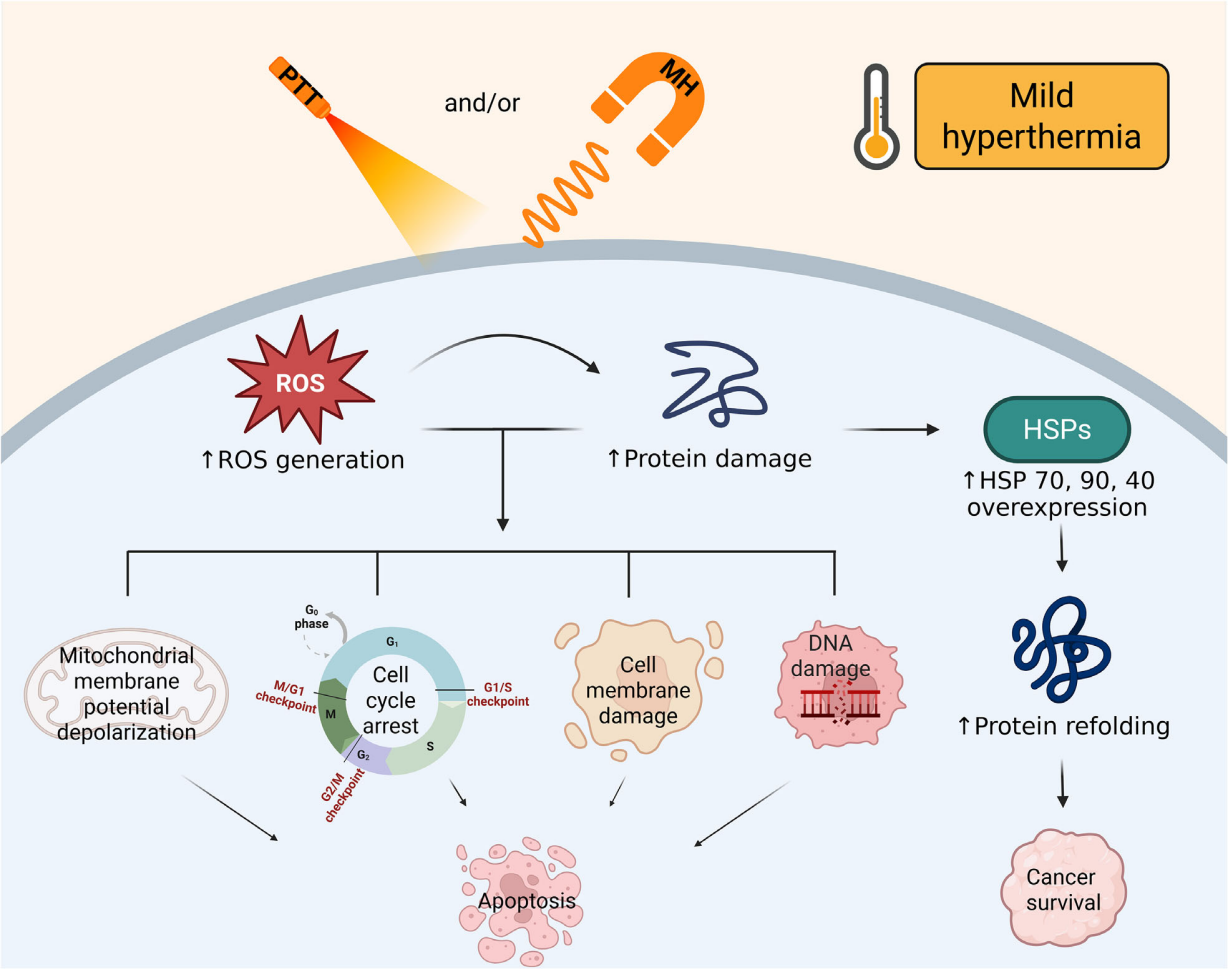
Intracellular effects of mild hyperthermia in cancer cells. Mild hyperthermia induces reactive oxygen species (ROS) generation and protein damage, which leads to several downstream effects, such as DNA damage and inhibition of DNA repair mechanisms, cell cycle arrest, mitochondrial membrane depolarization, and cell membrane damage. These processes collectively result in apoptosis or programmed cell death in cancer cells. Conversely, mild hyperthermia‐induced protein damage triggers the overexpression of HSPs, which repair or remove the damaged protein and promote protein refolding, enhancing cell survival and cancer cell regrowth.

Mild hyperthermia is defined as controlled heating of tumor tissue to non‐lethal or sublethal temperatures. The primary goal is not to induce a high degree of tumor cell death or extensive cellular damage, but rather to modulate the TME to sensitize the tumor tissue to secondary therapies, such as chemo‐ or immunotherapy [29, 168, 183, 184]. Compared to healthy tissue, tumors are more sensitive to increased temperatures, presumably owing to the low blood perfusion, resulting in poor heat dissipation, nutrient deprivation, hypoxic conditions, and low pH [184, 185]. A temperature of 45°C is commonly reported as the upper limit for mild hyperthermia applications in the literature [161, 170, 186, 187]. Whereas ablative treatments are applied only for a few seconds or minutes, mild hyperthermia conditions are normally maintained for prolonged periods of time, from a couple of minutes up to several hours [183, 188]. Several biological effects are induced by application of mild hyperthermia conditions, such as enhanced vascular perfusion and permeability, initiation of heat shock responses, and immune activation (Figure 6) [168, 189, 190]. Irreversible tissue damage, such as cell death and protein denaturation, typically occurs at temperatures higher than 42°C, depending on cell type and tumor physiology [168, 184, 189]. Rapid necrosis begins when temperatures reach 45°C [161]. In thermal ablation, tissue temperature is commonly increased to temperatures far exceeding 45°C, with the aim of ablating tumors by inducing irreversible cellular damage to the malignant tissue through coagulative necrosis. These elevated temperatures are typically maintained for only a few minutes, which is sufficient to induce protein denaturation, membrane disruption, and vascular occlusion [183, 189, 191]. Increasing the temperature further, up to 60°C, leads to nearly instantaneous cell death due to protein denaturation and melting of the plasma membrane [189].

Photothermal ablation has been explored as a standalone treatment with the goal of tumor eradication. However, ablative PTT is found to be associated with a risk of tumor recurrence and metastasis development [168, 183, 192]. Several preclinical studies have reported tumor destruction using ablative target temperatures, with no or limited tumor recurrence for at least 50 days post‐treatment after only 3–5 min of laser irradiation in tumor‐bearing mice [173, 193, 194, 195]. Despite these encouraging results, numerous studies to date have indicated that thermal ablation alone is often inadequate for effectively treating large tumors and eradicating untreated metastatic or distant tumor growth [29, 165, 183, 192]. An early study on NP‐mediated hyperthermia from 2003 demonstrated how combining a tumor target temperature of only 43°C with cytokine treatment resulted in tumor regression, while hyperthermia alone was insufficient to fully ablate malignant melanoma, exemplifying how secondary treatment strategies can leverage the beneficial physiological and cellular effects induced by mild hyperthermia [196]. Since this pioneering work, other studies have reported the use of mild or ablative hyperthermia for sensitizing tumors to additional therapeutic modalities. Indeed, by employing combinatory treatments, such as PTT combined with chemotherapy or immunotherapy, the limitations of individual therapies can be overcome, while the required dose for each treatment can be reduced to minimize off‐target toxic effects [197, 198].

Mild hyperthermia treatment of primary tumors triggers a range of beneficial structural and immunomodulatory responses, emphasizing its potential to enhance the anticancer effects of immunotherapy against both primary tumors and metastases. The physical effects observed with mild hyperthermia include increased perfusion and re‐oxygenation [101, 169, 170, 199]. and structural changes in the ECM [101, 170, 177, 178, 199, 200]. Moreover, several recent studies on preclinical murine models have demonstrated the ability of PTT to transform an immune‐excluded TME, which is characterized by a low presence of tumor‐infiltrating lymphocytes and antigen expression, into an inflamed tumor [170, 187, 201, 202, 203]. PTT‐induced immunomodulatory effects, such as immunogenic cell death and alleviation of tumor‐induced immunosuppression, contribute to creating an immunogenically hot TME, thereby enhancing the efficacy of immunotherapeutic approaches. Immunogenic cell death is a process where tumor‐associated antigens, damage‐associated molecular patterns, and pro‐inflammatory cytokines are released to activate the immune system. The damage‐associated molecular patterns promote the recruitment and activation of antigen‐presenting cells, which present the tumor‐associated antigens, activating T cells to eradicate tumor cells. As an example, the immune infiltrate of subcutaneously implanted breast tumors from immunocompetent mice treated by PTT mediated by gold‐decorated iron oxide nanoflowers administered intratumorally (average surface temperature maintained at 43°C for 15 min) was analyzed 12 days post‐irradiation [187]. A large increase in CD8^+^ T‐cell infiltration was observed, as well as higher levels of proinflammatory cytokines, highlighting the transition from a cold to a hot TME. A similar shift in the immune landscape was found in immunocompetent murine models of cholangiocarcinoma, where a combination of laser‐activated gold‐decorated iron oxide nanoflowers (average surface temperature 40–41°C for 15 min, repeated three consecutive days) and anti‐PD‐1 treatment resulted in reduced tumor stiffness and a favorable modulation of the immune microenvironment [203]. In particular, this study demonstrated collagen remodeling, an upregulation of immune‐potent cytokines, and a reduced presence of Tregs and immunosuppressive cancer‐associated fibroblast subsets as well as an increased infiltration and activation of dendritic cells and CD8^+^ T cells compared to controls, both in subcutaneously and more clinically relevant orthotopically injected tumors. Moreover, hyperthermia can induce cellular stress and partial necrosis, which upregulates the production of tumor‐specific antigens and HSPs by damaged or dying cells [170, 172, 184, 192, 196, 204]. The released tumor antigens can be captured by antigen‐presenting cells and used for priming and activation of endogenous effector T cells in draining lymph nodes [172, 187, 192, 204]. The release of damage‐associated molecular patterns from tumor cells additionally stimulates the production of proinflammatory cytokines and chemokines. For instance, 24‐h post‐light irradiation, elevated serum levels of proinflammatory cytokines (IL‐12p70, IL‐6, and TNFα) were found in tumor‐bearing mice, which can contribute to activation and antitumor activity of both endogenous and adoptively transferred T cells [192, 205]. In summary, PTT can trigger multiple processes that enhance both innate and adoptive immune responses against tumors, addressing some of the major hurdles to immunotherapy for the treatment of solid tumors, including the barriers posed by the hostile and immunosuppressive TME [21].

Physical barriers and immune evasion mechanisms within the TME hamper ACT infiltration and downregulate their antitumor activity. The presence of a dense and aberrant ECM, as well as a high interstitial fluid pressure and abnormal vasculature in the tumor, physically obstructs infiltration, distribution, and efficacy of therapeutic agents, including ACT. Moreover, solid tumors are protected by strong immunosuppressive barriers that impede effective immune responses [21]. The ability of PTT to induce a more immunogenic, T cell‐infiltrated TME characterized by an increased local and systemic expression of proinflammatory cytokines can significantly enhance the infiltration and activity of ACT [101, 169, 170, 192, 199, 206]. A pioneering study in 2013 demonstrated the power of combining PTT and ACT, showing that neither PTT nor ACT alone effectively eliminated secondary nor metastatic tumors in melanoma‐bearing mice [192]. Indeed, during the standalone gold NP‐mediated PTT, strong proinflammatory responses and an increase in immunosuppressive myeloid‐derived suppressor cell levels were observed, leading to accelerated lung metastasis growth and frequent primary tumor recurrence. Similarly, ACT alone had limited efficacy compared to non‐treated controls, presumably owing to the immunosuppressive TME. When used in combination, PTT significantly enhanced the antitumor effects of ACT, preventing recurrence and suppressing metastasis formation. In addition, PTT improved the antitumor effect of ACT on pre‐established metastases, despite PTT being applied only on the primary tumor, highlighting its systemic immunostimulatory impact.

Favorable effects of combining PTT and ACT compared to isolated treatments were also observed in a preclinical murine model (Raji tumor‐bearing mice), demonstrating the ability of PTT to attenuate the physical barriers to CAR‐T cell trafficking in the TME [170]. CD19‐specific CAR T cells were conjugated to ICG NPs by click chemistry between cyclooctyne moieties present on the NP surface and azido groups introduced on the CAR‐T cell surface via the intrinsic glycometabolism of azide‐glucose. After intravenous administration, the CAR T cells infiltrated efficiently into the PTT‐treated tumors, whereas a high accumulation was observed only at the periphery of the tumor in non‐irradiated control mice. These findings clearly demonstrate the effect of the physiological barrier in solid tumors in hampering CAR‐T cell infiltration and the TME modulation of PTT. The enhanced infiltration of CAR T cells in PTT‐treated tumors was likely due to efficient dilation of tumor blood vessels, enhanced blood perfusion, and degradation of collagen I fibers, resulting in a more permeable and less compact tumor structure. The mild photothermal treatment also stimulated the secretion of chemokines, resulting in an immune‐favorable TME and eventually tumor growth suppression. In another study, an enhanced infiltration of CD4^+^ and CD8^+^ CAR T cells in PTT‐treated solid tumors was observed in a melanoma‐bearing mouse model using immunocompetent mice [169]. To perform the treatment, tumor cells were subcutaneously injected in NSG mice on each flank, and only one tumor was subject to intratumoral injection of PLGA NPs loaded with ICG, followed by NIR light exposure. Preferential trafficking to and infiltration of anti‐CD19 CAR T cells in PTT‐treated tumors compared to contralateral controls demonstrated the ability of PTT to modulate the TME by dilating blood vessels, enhancing blood perfusion, destroying the ECM, and loosening compact tumor tissue. 20 days after the treatment, tumor growth was significantly inhibited, with tumor clearance observed in one‐third of the treated mice. Other strategies have combined PTT with the controlled release of immunomodulatory agents from NPs, such as chromium‐based NPs coated with a layer of a photothermal polymer, polydopamine, containing disulfide bonds, and loaded with a PI3K inhibitor to enhance CAR‐T cell therapy in solid tumors [206]. Cr^3^^+^ ions were released in the TME owing to the degradation of polydopamine facilitated by glutathione via the cleavage of the disulfide bonds in the polydopamine layer, especially in an acidic medium. In addition to the favorable pro‐inflammatory responses induced by PTT, the Cr^3^^+^ ions significantly promoted in vitro the migration of CAR T cells targeting B7‐H3 (also named CD276, overexpressed in many cancers, such as breast and liver cancer). In vivo, Cr^3+^ increased the expression of CXCL13 and CCL3 chemokines, promoting the intratumoral formation of tertiary lymphoid structures, which facilitated the infiltration of CAR T cells. The triple‐modality therapy was performed in a sequential regimen in immunodeficient NSG mice bearing a human hepatoma or PIK3CA‐mutated breast tumor: (i) intravenous injection of the NPs, (ii) PTT the following day, (iii) and intravenous CAR‐T cell infusion on day 3. The tumor‐bearing mice treated with the Cr‐based NPs and anti‐B7‐H3 CAR T cells showed efficient tumor regression following PTT, achieving a very high survival rate at 30 days, compared to only 40% of survival in the non‐irradiated chromium‐based NPs + anti‐B7‐H3 CAR‐T cell control group. This photo‐metallo‐immunotherapy strategy was enhanced by the incorporation of the chemotherapeutic agent alpelisib in the NPs, resulting in inhibition of tumor metastasis to the lungs. However, these results were generated in immunodeficient mouse models, which often inadequately represent clinical complexity. In another study, the catalytic properties of copper were leveraged to modulate the TME in combination with PTT and CAR‐T cell therapy against solid tumors [199]. Copper sulfide (Cu_2−x_S) NPs coated with hyaluronic acid, which can target CD44 overexpressed in many types of cancer, were used as photothermal agents and were able to generate ROS in the TME via the decomposition of hydrogen peroxide, present at a higher level in cancer cells compared to normal cells, into hydroxyl radicals. The production of ROS promoted the release of damage‐associated molecular patterns and induced partial tumor cell death, thereby enhancing the immunomodulatory effects of PTT and further supporting anti‐B7‐H3 CAR‐T cell recruitment and antitumor activity. Synergistic immunomodulatory effects were also observed by co‐encapsulating a TGF‐β inhibitor and ICG in hydroxyethyl starch‐polycaprolactone NPs for combined PTT and CAR‐T cell therapy towards solid tumors [101]. An intravenous injection of the NPs loaded with a TGF‐β inhibitor (LY) and ICG (LY/ICG@NP) enhanced the antitumoral activity of anti‐CD19 CAR T cells compared to anti‐CD19 CAR‐T cell treatment alone. Favorable antitumoral effects were also observed upon injection of ICG‐loaded NPs and NIR light irradiation (ICG@NP + PTT), which modulated the TME by increasing blood perfusion and reducing the density of the ECM by destroying tumor cells and ECM components, thereby enhancing the efficiency of anti‐CD19 CAR T cells. Whereas both LY/ICG@NP and ICG@NP + NIR treatments improved CAR‐T efficacy, the highest tumor volume inhibition rate was observed when combining the immunotherapeutic treatment with PTT (LY/ICG@NP + NIR), efficiently exploiting the favorable effects of each of the two TME modulation approaches.

Another immune‐evasion mechanism of solid tumors is associated with the downregulation of target antigen expression on tumor cells, resulting in a gradual loss of antitumoral activity and subsequent tumor regrowth over the course of the treatment. In a recent study in melanoma‐bearing mice (prepared by subcutaneous injection of melanoma cells in immunocompetent mice), adoptively transferred T cells exhibited high antitumor activity for the initial 9 days of treatment, while the survival rate was only 50% at day 21 due to antigen escape and tumor recurrence. [207] Employing adoptively transferred T cells loaded with gold NPs, NIR light irradiation was applied daily from day 9 to 15 after T‐cell injection to eradicate the resistant tumor cells and counteract tumor regrowth. PTT was initiated on day 9, when T cells started to lose control of tumor cell growth. Despite the negative impact of PTT on T‐cell viability, the combination treatment efficiently controlled the tumor growth, and mice treated with PTT posterior to ACT exhibited a very high survival rate on day 21. This study demonstrates that PTT can be used not only to modulate the TME to enhance T cell infiltration and activity, but also as a strategy to eliminate tumors that develop resistance to immunotherapy. Another important momentum is the advantage of loading NPs within tumor‐reactive T cells for highly efficient delivery of photothermal agents to the tumor site, resulting in accumulation of 20% of the injected NPs in the tumor, compared to less than 1% generally for intravenously injected free NPs [208], suggesting T cells as efficient carriers.

Despite numerous promising preclinical studies combining PTT with immunotherapy for partial or complete tumor eradiation, one must consider the simplicity of the employed preclinical murine models. Most studies have relied on subcutaneous xenografts in immunodeficient or ‐suppressed mouse strains, the most commonly used models for preclinical studies of CAR‐T cell therapies for solid tumors [209], which do not fully recapitulate tumor heterogeneity, the TME, nor relevant immune interactions. Orthotopic and/or immunocompetent models are comparatively underrepresented, limiting translational accuracy. To date, only a limited number of clinical trials have evaluated NP‐mediated PTT, using gold‐coated silica NPs for thermal ablation of head and neck, prostate, and lung cancers (e.g., AuroLase therapy) being the most prominent [210, 211], whereas no clinical studies combining PTT with immunotherapy have been reported. As the complexity of the models increases, new challenges related to toxicity, systemic effects, and the dose‐response relationships, both for the individual therapies and when used in combination, are expected. Bridging the gap between preclinical studies and clinical implementation will thus require further studies of delivery and retention of non‐toxic photothermal agents in tumors at distinct anatomical sites, followed by physiological clearance, consideration of NIR light penetration depth limits, interplay with the host immune system, and long‐term safety assessment in more advanced models.

Another major challenge is the lack of a standardized, transversal photothermal dose applicable across different photothermal agents and irradiation conditions, ideally derived from an understanding of in vivo dose‐response relationships. Particularly, the emergence of novel photothermal agents with distinct in vivo photothermal conversion efficiencies complicates cross‐study comparison. Consequently, a universal framework for quantifying therapeutic response across PTT parameters, including photothermal agent properties and dose, irradiation power and duration, and target time‐temperature profiles, is required. Progress towards standardization may be achieved through non‐invasive local temperature monitoring at the treatment site, enabling dose metrics based on time‐temperature profiles (e.g., CEM43°C), both for superficial and deep‐seated tumors. In this context, nanothermometers, which are nanoprobes capable of real‐time surface‐proximal temperature monitoring, represent a novel and promising approach for real‐time intratumoral temperature assessment if co‐delivered with photothermal agents [212].

Overall, an increasing number of preclinical studies demonstrated that PTT can effectively trigger specific antitumor and proinflammatory immune responses that complement ACT. Using organic and inorganic photothermal agents with or without additional immunostimulatory agents, PTT helps modulate the TME in mouse models. This modulation includes breaking down physical barriers and increasing tumor immunogenicity, thereby converting immunologically cold tumors into hot ones. As a result, PTT addresses several key challenges that limit the effectiveness of ACT in solid tumors [21]. However, further studies are necessary to clarify and control the exact mechanisms of immunomodulation induced by PTT to fully understand and exploit the synergistic effects obtained in combination with ACT, also in more complex preclinical models. Nonetheless, PTT remains a highly active research topic in nanomedicine research for cancer therapy, and further insights into the mechanisms and interplay between PTT and immunotherapy, long‐term effects and safety, and dose‐response relationships could contribute to the translation from preclinical studies to the clinic.

### 3.2 Adoptive Cell Therapy Combined with Magnetic Hyperthermia

Similar to PTT, MH is a localized thermal therapeutic strategy that can remodel the TME and boost the efficacy of immunotherapies by immunogenic cell death [213, 214]. In the presence of an alternating magnetic field, magnetic NPs generate localized heat that stimulates tumor cell death [215]. In addition, iron oxide NPs exhibit peroxidase‐like activity, converting hydrogen peroxide in the TME into ROS that induce tumor cell death [216]. Nanoscale heating from MH amplifies this activity, thus elevating intracellular ROS levels and triggering oxidative stress that destroys tumor cells [217]. This synergy between localized heating and enhanced ROS generation is crucial for inducing immunogenic cell death and stimulating a robust immune response, even in hypoxic tumor conditions. Moreover, magnetic NPs can selectively accumulate in the TME through a combination of passive targeting via the EPR effect and active targeting with specific ligands. Compared to other thermal ablation methods, such as laser irradiation or radiofrequency, MH offers deeper tissue penetration and reduced disruption from surrounding tissues due to targeted accumulation and localized heating [218, 219].

While PTT is limited by the penetration depth of the laser, MH does not have a penetration limitation. However, large doses of magnetic NPs are usually required to increase the temperature substantially [220], which might induce toxicity issues. Additionally, the presence of lesions in different organs at varying depths can limit the penetration and effectiveness of the alternating magnetic field. Consequently, MH alone is generally found to be inefficient towards targeting multiple and metastatic tumors. As a result, the combination of MH with other therapies, primarily chemotherapy or immunotherapy, has advanced rapidly [221, 222]. These combination therapies have not only achieved efficient tumor ablation with lower magnetic field strength and reduced drug dosage, but have also demonstrated significant advantages in addressing metastatic and multiple tumors [223, 224, 225]. For example, combination therapy based on MH and immune checkpoint blockade (α‐PD‐L1 antibody) was reported to prevent tumor metastasis by enhancing cytotoxic T lymphocyte infiltration, thereby reducing mortality in a breast cancer mouse model using magnetic NPs [226]. In another work, iron oxide nanorings were used for MH in combination with anti‐PD‐L1 immunotherapy, inducing immunogenic cell death, preventing lung metastases, and inhibiting distant tumor growth with no toxicity in other organs [227].

The combination of MH with ACT represents an encouraging therapeutic strategy, as MH can improve ACT efficacy by enhancing immune cell infiltration, triggering immunogenic cell death, and altering the immunosuppressive TME. Dendritic cells are professional antigen‐presenting cells that induce adaptive immunity by activating both cytotoxic and helper T cells, and they also contribute to innate immune responses by activating NK cells. Dendritic cell therapy stimulates the patient's own immune system by activating endogenous immune cells, including T cells, and initiates an antitumor immune response, rather than relying on the infusion of external engineered immune cells, such as CAR T or TCR T cells [228, 229]. Moreover, dendritic cell therapy carries a very low risk of cytokine release syndrome or on‐target/off‐tumor toxicities, making it a relatively safer approach compared to CAR T cells. Importantly, dendritic cell therapy can induce long‐term immune memory, which enhances its potential as an effective cancer treatment. However, some limitations remain, such as limited clinical efficacy in solid tumors due to the immunosuppressive TME, as well as manufacturing complexity and high cost. These challenges highlight the need for combinatorial strategies, such as combining dendritic cell therapy with MH, to improve therapeutic outcomes. In this regard, the efficacy of MH mediated by liposomes encapsulating magnetic NPs, followed by immature dendritic cell therapy, was investigated in a melanoma preclinical mouse model [230]. In vitro, MH induced cancer cell death and release of Hsp70, which triggered dendritic cell maturation, resulting in enhanced antigen presentation and migration to lymph nodes. The combination treatment administered intratumorally in melanoma tumor‐bearing mice (prepared by subcutaneous injection of melanoma cells in immunocompetent mice) exhibited tumor regression in 60% of the mice, and this outcome was not observed for the MH or dendritic cell therapy group alone. This study demonstrates the effect of combination therapy with durable antitumor immunity, which could have future application in advanced melanoma patients. Importantly, it shows that intense hyperthermia can enhance dendritic cell maturation through necrotic cell death and HSP expression, reinforcing its role in in situ vaccination. In another study, the same group provided evidence of the therapeutic potential of MH mediated by the magnetic liposomes in combination with dendritic cell immunotherapy in a mouse model of EL4 T‐lymphoma (prepared by subcutaneous injection of a cell line isolated from a chemically‐induced lymphoma, in immunocompetent mice) [231]. The liposomes were injected into the EL4 nodules and exposed to an alternating magnetic field to reach 45 °C for 30 min twice at 24‐h intervals. After hyperthermia, immature dendritic cells were injected into the tumor, resulting in tumor regression in 75% of mice compared to only 12.5% treated by MH only.

In conclusion, when combined with ACT, MH has shown high efficacy across different tumor models, particularly by enhancing immune cell infiltration and inhibiting tumor progression. The localized heat generated by MH not only induces tumor cell death but also stimulates the release of cytokines and HSPs, which promote dendritic cell maturation and create a more immunogenic TME. An advantage of MH compared to PTT is the absence of penetration depth limitations, making it applicable to tumors inaccessible to light‐based approaches. Nevertheless, the number of preclinical studies combining MH and ACT remains limited. Given the promising results from studies on MH as a monotherapy and its capacity to remodel the TME, more efforts should be made to integrate MH with ACT to improve the efficacy against solid tumors.

Building on the synergistic effects of NP‐mediated hyperthermia with ACT, attention has increasingly turned to strategies that further enhance the precision and efficacy of immune cell delivery. One promising approach involves the magnetic targeting of engineered T cells, which exploits magnetic NPs and an external magnetic field to guide immune cells directly to tumor sites.

## 4 Magnetically Targeted Engineered T Cells

Efforts to address the challenge related to poor CAR‐T cell infiltration in solid tumors can be broadly categorized into two strategies: (a) engineering T cells to modulate the TME, such as by expressing chemokine receptors or secreting cytokines to enhance infiltration (Section 2), and (b) using physical tools to disrupt the physical barrier of the TME, for example by PTT or MH (Section 3), and guide cells to the tumor site using an external magnetic field [232, 233]. Magnetic targeting involves loading CAR T cells with magnetic NPs, such as iron‐based NPs. This approach has the advantage of not only enhancing tumor homing but also allowing non‐invasive imaging through MRI.

For instance, superparamagnetic iron oxide NPs were used to magnetize CAR T cells expressing the CAR‐specific antigen chondroitin sulfate proteoglycan (CSPG4), primarily expressed on melanoma cells [234]. For this purpose, T cells after electroporation with mRNA were incubated with the NPs. Electroporation of the T cells increased NP loading. Transmission electron microscopy showed the localization of the NPs on the plasma membrane and within vesicles. However, the presence of the NPs reduced the CAR surface density and decreased CAR‐T cell killing at lower effector:target ratios. The NP‐loaded CAR T cells released lower amounts of pro‐inflammatory cytokines, which correlated with reduced cytotoxicity. Due to the reduced cytokine release, T cells might also secrete fewer damage‐associated molecular patterns, thereby shifting the induced cell death from pyroptosis (which contributes to CAR‐T‐related cytokine release syndrome) to apoptosis (which is less toxic). The magnetized CAR T cells could be guided and accumulate in tumor spheroids under dynamic flow conditions to simulate blood circulation, and were detected by MRI, suggesting potential for in vivo navigation and monitoring. Alternatively, TCR T cells (specific for the melanoma antigen MelanA or the endogenous TCR specific for the cytomegalovirus antigen pp65) were also modified with superparamagnetic iron oxide NPs by electroporation, demonstrating an enhanced tolerance to this type of modification compared to CAR T cells [235]. Indeed, functional testing confirmed that the loading of NPs in the cells did not impair either exogenous or endogenous TCR activity, as shown by flow cytometry‐based cytotoxicity assays with the cognate antigen. TCR T cells also retained cytokine secretion, degranulation, and antigen‐specific cytotoxicity, possibly due to their lower activation threshold requiring only one peptide‐MHC for activation.

In another approach, iron oxide NPs were internalized by incubation into the endolysosomes of EGFR‐targeted CAR T cells without compromising their viability, CAR expression, and cytotoxic function [236]. The application of an external magnet placed over the tumor significantly enhanced the recruitment of the magnetic CAR T cells to the tumor in mice bearing subcutaneous EGFR‐expressing human lung cancer cell‐derived xenograft, resulting in tumor growth inhibition. An enhanced CAR‐T cell accumulation was observed by MRI at the tumor site three days post‐injection, with an ex vivo analysis showing a markedly greater tumor infiltration with iron oxide‐labeled CAR T cells compared to non‐labeled ones. Moreover, the NP‐loaded cells allowed real‐time tracking by MRI, offering a potential companion diagnostic tool. While promising for magnetic guidance of CAR T cells, this strategy requires further validation in immunocompetent models, as current preclinical studies in syngeneic systems show only transient CAR‐T cell persistence, unlike the prolonged survival observed in immunodeficient mice.

This limitation was overcome in a study where Fe_3_O_4_ nanoclusters were functionalized with anti‐PD‐1 antibodies via a pH‐sensitive linker (imine) to facilitate their conjugation to effector T cells thanks to their PD‐1 expression [122]. The magnetic T cells were guided to the tumor using an external magnetic field, enabling reduced dosing frequency and lower systemic toxicity. In an immunocompetent murine model (E.G7‐OVA lymphoma‐bearing mice), the T cells decorated with the magnetic nanoclusters significantly inhibited tumor growth. Notably, this approach was successfully extended to tumor‐infiltrating lymphocytes, which similarly suppressed tumor proliferation and prevented metastasis.

Magnetic propulsion enables long‐distance delivery with precise spatial targeting, but it typically lacks the physical force required to penetrate dense tumor tissue. In contrast, focused ultrasound can generate localized biophysical effects that propel targets deep into tissue (beyond 10 cm), yet its effectiveness is limited to short distances because acoustic energy dissipates within biological tissues [237]. In this regard, a precision immunotherapy strategy has been developed using magnetic‐acoustic sequential actuation to enhance anti‐CD19 CAR‐T cell infiltration into solid tumors [238]. In this two‐step approach, CAR T cells were functionalized with iron oxide NP‐containing immunomagnetic beads via click chemistry. First, an external magnetic field was used to guide the magnetically responsive cells through the bloodstream to the peritumoral region. Then, focused ultrasound power using acoustic tweezers applied localized mechanical forces that propelled the cells deep into the dense tumor tissue. In immunodeficient mice (CD19‐SPCA1 (a human lung carcinoma cell line) tumor‐bearing mouse model), this method increased intratumoral CAR‐T cell accumulation 6.5‐fold compared with unmodified cells, and reduced the proportion of exhausted T cells by 88%. The magnetic CAR T cells effectively reduced tumor growth and prolonged mouse survival. While promising, clinical translation may be limited by the need for specialized magnetic and acoustic equipment and the manufacturing complexity of reproducibly producing bead‐coated CAR T cells.

Overall, magnetically guided delivery of adoptively transferred T cells offers strong potential to improve the efficacy and safety of ACT for solid tumors by enhancing tumor‐specific accumulation, reducing off‐target toxicity, lowering therapeutic cell doses, and enabling real‐time MRI tracking of cell biodistribution. Nonetheless, significant technical challenges remain. Due to their small size, high nucleus‐to‐cytoplasm ratio, and non‐phagocytic nature, T cells have limited capacity for NP internalization, making surface conjugation more feasible than intracellular loading. Moreover, attachment of NPs on the cell surface can impair CAR expression and function, an effect not seen in TCR‐engineered T cells, posing a challenge given that CAR T cells require both high CAR density and abundant target antigens for effective cytotoxicity. Clinical translation is further hindered by the need for uniform manufacturing of magnetic NP‐decorated cells with consistent NP loading, the requirement for specialized magnetic guidance equipment not available in oncology clinics, and further studies to clarify the long‐term biocompatibility, fate, and biodegradation of magnetic NPs. Finally, as these products combine living cell therapy with NPs, they would likely face more complex regulatory pathways.

Having explored strategies for magnetically guiding engineered T cells to tumor sites, the focus now shifts to approaches that enable precise control over their activation once in vivo. NP‐mediated systems offer the ability to generate and remotely activate CAR T cells within the body, representing a step towards more precise, effective, and safer ACT.

## 5 In vivo Nanoparticle‐Mediated Generation and Controlled Activation of CAR T Cells

**FIGURE 7:**
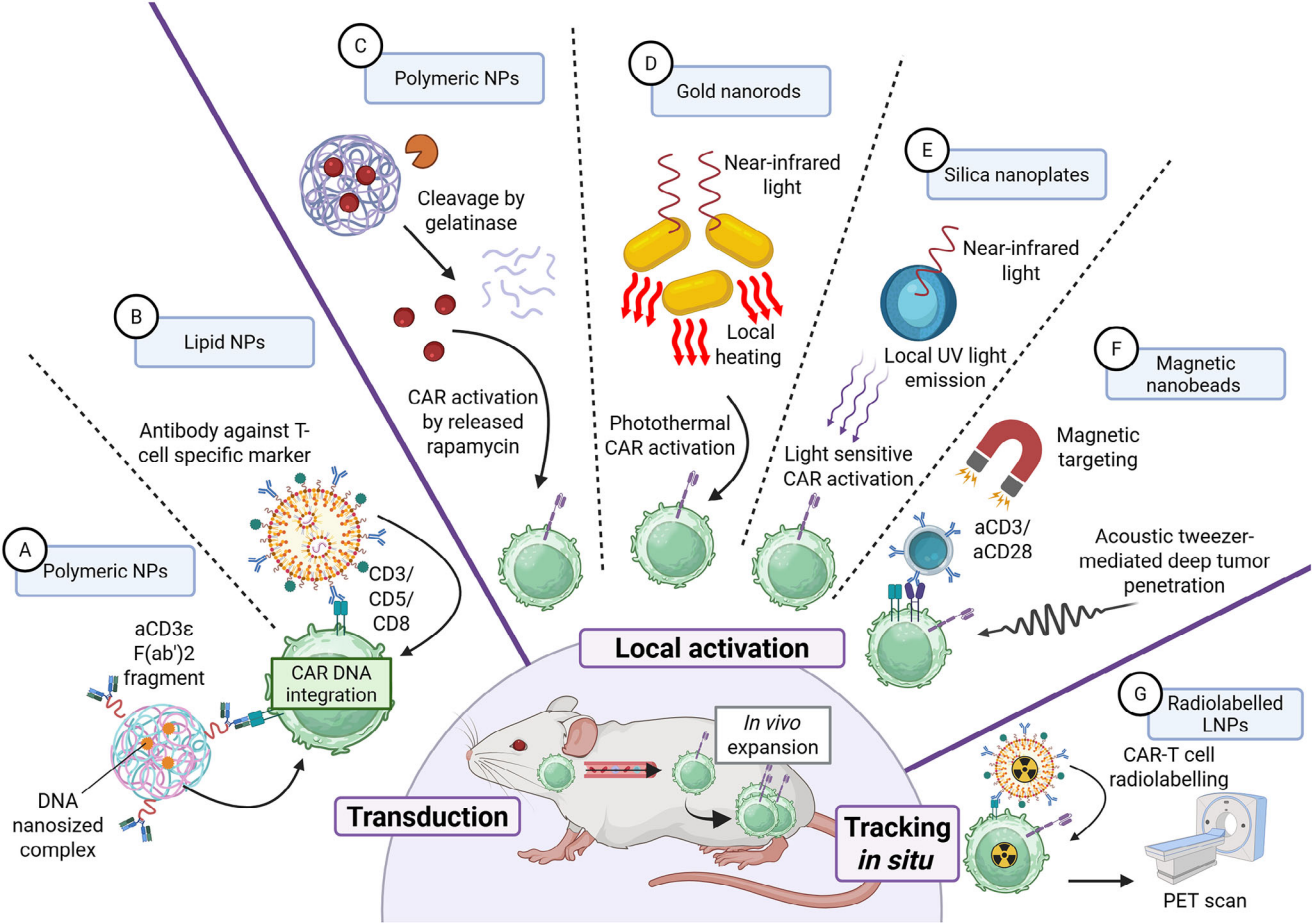
Overview of NPs used for the in vivo generation and controlled activation of CAR T cells. Different types of NPs, such as (A) polymeric NPs and (B) LNPs, were developed to efficiently transduce T cells in vivo. For controlled activation of CAR T cells in situ, NPs were used (C) as carriers for the selective delivery of rapamycin to trigger rapamycin‐sensitive switchable CAR activation, (D) to generate local heat under NIR light irradiation for thermal activation of CAR T cells, (E) to produce UV light upon NIR irradiation to activate light‐inducible CAR T cells, or (F) for local CAR‐T cell activation after magnetic targeting. (G) In situ tracking was achieved by PET/CT imaging using LNPs loaded with radioactive PSMA.

Some major drawbacks of CAR‐T cell therapies include ex vivo manufacturing complexities, high cost, and adverse events such as cytokine release syndrome. The classical process relies heavily on individualized ex vivo cell manipulation, including leukapheresis, gene editing, viral transduction, and expansion, which limits scalability and timely treatment [239, 240]. Several strategies rely on viral vectors to directly and efficiently target T cells in vivo for modification into CAR T cells. However, despite being attractive for their efficiency and long‐term effects, viral therapies have been shown to generate non‐negligible side effects in patients [240]. Innate immune sensing of capsids or other viral components can lead to harmful immunogenicity by activating plasmacytoid dendritic cells in murine settings [241]. Viral vectors strategies could hypothetically drive such an effect in humans based on clinical observations [240], resulting in some hurdles in clinical development. Non‐viral in vivo generation of CAR T cells has emerged as a novel alternative to tackle these challenges [242]. It aims at modifying T cells in situ for their efficient and specific redirection to the tumor. In this regard, the use of NPs could significantly improve overall safety, specificity, and efficiency by enabling direct T cell reprogramming within the patient. Figure 7 gives an overview of different strategies using NPs for in vivo generation and controlled activation of CAR T cells.

The main strategies currently developed for in vivo generation of CAR T cells are based on polymeric NPs, LNPs, or exosomes. Cationic polymeric NPs, including polyethyleneimine, polyamidoamine, or poly (β‐amino ester) (PBAE), mixed with DNA molecules can form electrostatic complexes that facilitate cellular uptake. These polymer‐DNA complexes are used to deliver CAR transgenes into T cells, offering a non‐viral alternative for gene expression, and can be coformulated with adjuvants or surface‐modified to improve targeting and endosomal escape. Polyethyleneimine is already used in clinical trials for DNA‐based gene therapies [243]; however, most approaches are based on PBAE, as it shows promising in vivo results [244]. For instance, PBAE NPs loaded with anti‐CD19 CAR were developed to target B cell leukemia (Figure 7a) [245]. Specifically, PBAE was functionalized with a peptide containing a microtubule‐associated sequence and a nuclear localization signal to enable rapid nuclear import of the genetic material through the microtubule transport system. The polymer was mixed with a plasmid DNA encoding a leukemia‐specific CAR, leading to the condensation of DNA into nanosized complexes. T cell‐targeting anti‐CD3ε F(ab′)2 fragments were covalently conjugated to polyglutamic acid, which was adsorbed on the NP surface via electrostatic interactions. The in situ programmed CAR T cells accumulated in treated leukemia‐carrying mice, resulting in significant tumor regression. A principal limitation of this work was the restricted capacity of the DNA NPs for CAR gene loading. In addition, the persistence of CAR expression in vivo was not considered, making the translation of this strategy into patients still challenging. In another study, the same group used PBAE NPs loaded with a CD19‐specific CAR and coated with an anti‐CD8 antibody to target cytotoxic CD8^+^ T cells [246]. The reprogrammed T cells induced regression of leukemia and prostate cancer in vivo with similar efficacy as ex vivo produced adoptive T cells. Unlike DNA‐based nanocarriers, mRNA molecules have the advantage of being translated into therapeutic proteins directly in the cytoplasm, bypassing the need for nuclear entry and enabling a rapid onset of therapeutic action. The use of mRNA‐based strategies is currently gaining interest compared to DNA‐based ones to limit safety issues. Due to its transient nature, mRNA‐based CAR expression has been shown to reduce side effects [52]. For CAR T cells, it has been shown that expression mediated by mRNA‐LNPs could lead to less exhausted T cells compared to lentiviral‐based strategies [247]. The absence of exhausted CAR T cells would generate a superior response against the tumor compared to CAR T cells manufactured through DNA approaches. The transient nature of CAR mRNA expression offers the advantage of limiting on‐target/off‐tumor effects, especially when mRNA is delivered locally by NPs, such as LNPs, diminishing adverse effects in patients and offering improved clinical care. Nevertheless, there are limitations to mRNA‐based approaches. Indeed, transitory approaches limit the persistence of engineered T cells in patients and require multiple injections to generate a clinical effect. However, this absence of persistence has not been described in DNA‐based strategies. The use of NPs (e.g., LNPs) or viral vectors to deliver DNA may favor the establishment of a long‐term memory response that could reduce potential risks of relapses. mRNA methods also failed to successfully control the development of certain solid tumors in some cases [248]. However, using NPs combined with mRNA transduction might be of interest to ensure continuous mRNA supply locally for CAR‐T cell persistence and to prevent relapse, while limiting side effects. It is also to be noted that mRNA‐based CAR manufacturing appears more cost‐effective and scalable, placing it as a valuable strategy for the clinic.

The most promising approaches using NPs for in vivo generation of CAR T cells employ LNPs loaded with DNA or RNA, which can outperform mRNA electroporation approaches for transfection efficacy [247]. LNPs present major advantages such as high encapsulation efficiency of nucleic acids, low immunogenicity and biocompatibility, scalability, and good manufacturing practice‐compatibility. One of the seminal studies demonstrating in vivo CAR delivery using LNPs was designed by targeting CD5 for the generation of CAR T cells against the fibroblast activation protein in a model of heart failure, demonstrating significant reduction of fibrosis and restored cardiac function in treated animals (Figure 7b) [249], given that T cells and a small population of B cells naturally express CD5, which is not essential for T cell effector activity [250], This study is the first showing impressive results, yielding 20–80% CAR conversion in mice, underscoring the targeting potential of the LNPs beyond cardiac diseases, especially in oncology. Other approaches targeting CD3 have shown promise. Indeed, LNPs modified with a CD3 antibody and loaded with a plasmid containing the combination gene of IL‐6 short hairpin RNA and anti‐CD19 CAR were used to specifically target T cells via CD3 antibody‐mediated recognition in leukemia NSG mouse model, and to efficiently generate anti‐CD19 CAR T cells with IL‐6 knockdown [251]. This strategy enabled selective elimination of CD19‐overexpressing leukemia cells while mitigating IL‐6‐induced cytokine release syndrome. Recently, CD8‐targeted LNPs were designed to deliver anti‐CD19 CAR mRNA to CD8^+^ T cells and CD8^+^ NK cells [252]. A rapid CAR expression (within hours) was observed, along with effective tumor and B cell depletion in humanized leukemia xenograft mice, as well as transient, manageable expression in cynomolgus monkeys. The authors showed that cytotoxic function was mostly exerted by CD8^+^ T cells, as depletion of NK cells did not induce a significant effect on B cell lysis. Joint transduction of CD8^+^ T cells and CD8^+^ NK cells might lead to higher off‐target effects, but a better immunological response might be reached against hard‐to‐target solid tumors, for example. In another study, the potential of virus‐mimetic fusogenic nanovesicles bearing an anti‐CD19 CAR protein was demonstrated for in vivo generation of anti‐CD19 CAR T cells [253]. A T cell fusogen was designed by adding an anti‐CD3 single‐chain variable fragment to reovirus or measles fusogen to redirect the fusion to T cells. No toxicity was observed in a syngeneic model of B cell lymphoma, while inhibiting the growth of the tumor. An additional advantage of this approach is that transient CAR protein expression without genomic interference may reduce tumorigenesis risk. Notably, no comparative data using virally modified T cells were presented in this study, making it difficult to assess the full potential of this approach. In addition, this strategy may be unsuitable for patients with impaired T cell function, where conventional CAR‐T cell therapy remains appropriate. Overall, owing to their scalability and versatility, LNPs seem to be the most promising NPs for in vivo CAR‐T cell generation. Numerous clinical trials are ongoing with LNPs functionalized with CARs targeting different antigens, such as TROP2, CD19, and GPC3 [254, 255, 256, 257].

Interestingly, mRNA encoding BiTEs has also been encapsulated in LNPs [258]. In this context, the liver was used as a bioreactor for the release of the therapeutic proteins; this is advantageous in terms of delivery since hepatic cells are easier to target than T cells [259].

Facing other challenges as on‐target, off‐tumor toxicity, innovative approaches for “on‐tumor only” activation of CAR T cells have been reported, as an attempt to reduce side effects in patients. For example, gelatinase‐responsive polymeric NPs were developed for the selective delivery of rapamycin to activate a rapamycin‐sensitive heterodimerizing switch (Figure 7c) [260]. In the design of CAR T cells, a switch generally refers to a separated controllable domain built into the CAR construct system that will grant full CAR signaling upon activation by an exogenous signal. It allows T cell activity regulation in a more precise, reversible, and safer way. Gelatinases are matrix metalloproteinases and are highly expressed in gastric cancer. In this work, polymeric NPs loaded with rapamycin, used as a switch, were prepared from a methoxy PEG‐polycaprolactone copolymer bearing a gelatinase‐cleavable peptide between the two segments. In contact with gelatinase, this nanomodified switch released rapamycin that triggered CAR signaling. This approach improved overall safety in mice bearing a human gastric adenocarcinoma cell line, avoiding cytokine release syndrome and reducing on‐target/off‐tumor effects with inactivated CAR T cells exerting minimal effects on normal cells. Another strategy for “on‐tumor only” activation of CAR T cells was based on the use of gold nanorods to induce photothermal (40‐42°C) activation of anti‐CD19 CAR T cells to produce transgenes that enhance their function [261]. To impart heat responsiveness, T cells were engineered with thermal gene switches composed of heat‐shock elements and core promoters (Figure 7d). These thermal constructs were designed to regulate a range of immunostimulatory genes. The data showed localized activation of CAR T cells in mice and enhanced T cell functions, including proliferation and tumor targeting, leading to an efficient tumor regression compared with the adoptive transfer of T cells alone. An alternative method explored light‐switchable CAR in CD8^+^ T cells (Figure 7e) [262]. Functional domains of the CAR were split inside the cells and paired with light‐sensitive modules on each part. Signaling occurred only upon both antigen recognition and localized UV light, generated by silica‐coated upconversion nanoplates administered directly into the tumor and activated by NIR light. The nanoplates were surgically removed after the treatment by imaging‐guided surgery. A significant improvement in anti‐tumor response was demonstrated against CD19^+^ melanoma cells compared to CD19^−^ tumors with an effect only at the site of light exposure. This study established a nano‐optogenetic immunomodulation platform enabling spatiotemporally controlled activation of T cells. A similar strategy using upconversion NPs was reported for remote control of calcium signaling and the immune system during dendritic cell‐based immunotherapy [263]. In another work, an elegant approach relies on anti‐CD19 CAR T cells decorated with immunomagnetic beads by click chemistry (Figure 7f) [238]. The beads were composed of iron oxide NPs functionalized with T cell activators (anti‐CD3 and anti‐CD28 antibodies) to mimic antigen‐presenting cells and stimulate the proliferation and activation of CAR T cells in the tumor. The CAR T cells were magnetically directed to the tumor site, and a second step of acoustic penetration was performed to allow deep tumor penetration, resulting in in situ CAR‐T cell activation and expansion. Compared to regular CAR‐T cell treatment in mice, this approach led to a higher infiltration of cytotoxic CD8^+^ CAR T cells at the tumor site. Interestingly, the magnetic CAR T cells expressed fewer exhaustion markers and showed significantly higher expression levels of cytokines (IL‐2, TNF‐α, and IFN‐γ) compared to conventional CAR T cells.

Finally, monitoring CAR‐T cell therapy in situ is crucial for tracking the generation and migration of engineered T cells, enabling real‐time assessment of therapeutic progression. In this context, prostate‐specific membrane antigen (PSMA) mRNA and anti‐CD19 CAR mRNA were loaded in anti‐CD5‐conjugated LNPs, enabling tracking of in situ‐engineered CAR T cells by positron emission tomography/computed tomography (PET/CT) imaging using a radioactive PSMA that binds to the expressed PSMA protein (Figure 7g) [264].

Overall, the strategies presented have demonstrated promising results for in vivo generation of CAR T cells in a safe, localized, and efficient manner, mostly in the context of mRNA delivery. However, the majority of the studies discussed here report promising findings from mouse models. As previously mentioned, clinical trials are ongoing mostly using LNPs, but, to date, further patient‐based evidence is still needed to assess the potential of these strategies. It is also to be noted that the impact of NPs on T cell metabolism remains poorly understood and might be detrimental for the generation of viable CAR T cells in vivo. Most studies do not fully address toxicity issues that could impact the potential of a given strategy for clinical applications. Growing efforts to overcome the limitations of CAR‐T cell therapy could benefit from the immunomodulatory properties of NPs and exosomes, which have emerged as promising adjuncts to enhance therapeutic precision and control adverse effects [265]. The integration of engineered exosomes with CAR‐T cell therapy would enable novel, cell‐free immunotherapeutic approaches with enhanced targeting, safety, and manufacturing scalability. NPs can be designed to modulate T‐ cell metabolism as a potential immunotherapeutic strategy [266]. It would be relevant to use NPs as metabolic modulators for in vivo CAR‐T cell generation, as enhancing T cell metabolism and mitochondrial fitness are critical for improving ACT persistence in solid tumors. Recently, another group has also presented a pharmacokinetic model supporting the introduction of this method for further clinical trials [267]. Despite translational and regulatory challenges, preclinical successes and modular engineering platforms position exosome‐mediated therapies as a transformative frontier in next‐generation cancer immunotherapy [268].

While NP‐mediated approaches enable in vivo generation and controlled activation of CAR T cells, achieving precise targeting remains a critical challenge. To further enhance delivery efficiency and specificity, strategies involving the coating of NPs with engineered cell membranes have emerged. This biomimetic approach allows for improved circulation and tumor targeting.

## 6 Engineered Cell Membrane‐Coating of Nanoparticles to Enhance Targeting Efficiency

Efficient targeting of cancer cells or other cells present in the TME, such as cancer‐associated fibroblasts and tumor‐associated macrophages [269], is of utmost importance when designing NPs for cancer treatment. The validity of passive targeting via the well‐known EPR effect has been increasingly questioned [42], suggesting that active targeting may be necessary to ensure sufficient NP accumulation in tumors. Beyond improving efficacy, targeting also reduces the accumulation of NPs in healthy tissues and organs, thus reducing toxicity. Inspired by how cells interact with their surroundings via receptors and antibodies, a common strategy for active targeting is to conjugate targeting molecules onto the NP surface. Additionally, for the NPs to reach the tumor site, they must avoid opsonization and subsequent phagocytosis by immune cells. The gold standard for this has been to conjugate biocompatible molecules such as PEG [270]. However, anti‐PEG antibodies have been detected in humans both before and after exposure to PEG, which accelerates the clearance of PEGylated NPs and can potentially cause adverse effects [271]. An alternative approach for targeting that has sparked the interest of many researchers is to directly coat the NPs with cell membranes [272, 273]. The cell‐membrane‐coated NPs can inherit certain functions of the source cell, including its ability to recognize certain targets in the body [274]. This is particularly valuable for active targeting, as it can increase the accumulation in the target tissues. Indeed, the cell membrane contains diverse components (proteins, lipids, receptors, ligands, and sugar moieties) that enable targeting (e.g., CAR) and immune evasion (e.g., CD47). For instance, cancer cell membranes are being studied for this application to enable homologous targeting, a self‐recognizing ability of cancer cells [275, 276]. In addition to their targeting ability, cell‐membrane‐coated NPs display higher stability in physiological conditions and increased blood circulation time, for instance, compared to PEGylated NPs. It was shown that iron oxide NPs coated with cell membranes derived from red blood cells had a significantly increased circulation time compared to their PEGylated counterparts [277]. This is attributed to the intrinsic stealth properties, high biocompatibility, and negligible immunogenicity of cell membranes, in addition to the presence of the “don't eat me”‐marker CD47.

To make the targeting even more specific, the source cells can first be genetically modified to express certain proteins on the surface that can recognize specific targets. In the last couple of years, several studies have emerged using engineered cell membranes, such as those from CAR T cells, to coat NPs [278, 279, 280, 281, 282]. These cells are engineered to recognize specific tumor‐related antigens, and the resulting membrane‐coated NPs have an increased tumor accumulation. In most of these studies, the NPs have been loaded with anticancer drugs or display photothermal properties, and the engineered cell membrane‐coated NPs are used for combined ACT and chemotherapy or PTT.

In one study, mesoporous silica NPs loaded with IR780, a NIR fluorophore and photothermal agent, were coated with CAR‐T cell membranes targeting glypican‐3, which is overexpressed in hepatocellular carcinoma cells [281]. CAR‐T cell membrane‐coated NPs outperformed both uncoated and non‐engineered T cell membrane‐coated NPs in targeting efficiency in vitro and in vivo. When combined with laser irradiation, the CAR‐T cell membrane‐coated NPs inhibited tumor growth in a preclinical model of hepatocellular carcinoma, demonstrating the potential of using CAR‐T cell membrane‐coated NPs in cancer treatment.

Another aspect of using engineered‐cell membrane camouflage that makes it an even more powerful strategy is the possibility of developing dual targeting. For example, a treatment for glioblastoma recurrence consisting of NPs with photothermal properties coated with T cell membranes engineered to express two different receptors was developed [280]. One of the receptors could target the epidermal growth factor receptors on glioblastoma cells, and the other CD133 receptors expressed by glioblastoma stem cells. The NPs were stable in PBS, cell culture medium, and mouse blood for 16 days at 4°C. A hematological analysis performed 14 days after intravenous injection showed no significant difference from controls. Another strategy for dual targeting relies on utilizing hybrid membranes, where cell membranes from two different source cells are mixed for coating NPs. For instance, an approach to decorate NPs was developed by combining membranes from tumor cells that overexpress ovalbumin and T cells overexpressing PD‐1 [282]. The NPs were mainly cleared via hepatic phagocytosis, with most being eliminated from the body within 6–8 h. This strategy increased the targeting capability of the NPs while simultaneously improving antigen presentation via ovalbumin, stimulating an in vivo immune response.

Beyond CAR T cells, similar approaches using other engineered immune cells have shown promising results [283, 284]. Different cell membranes have different surface receptors and ligands that can be exploited together with engineered receptors to enhance tumor accumulation. In one study, rapamycin‐loaded PLGA NPs were coated with macrophage cell membranes expressing PD‐1 to treat glioblastoma [283]. Here, the tumor targeting was attributed to the ability of macrophages to home to the glioblastoma microenvironment, whereas the engineered receptor had a therapeutic effect, as it acted as an ICI. In another study, micelles that could release a ferroptosis inducer upon laser irradiation were coated with CAR‐NK‐derived exosomes to treat HER2^+^ breast cancer brain metastasis [284]. The CAR was designed to recognize HER2^+^ tumor cells, and exosomes were chosen because they contain surface ligands that can interact with the cerebral vascular endothelial surface receptors to facilitate crossing of the blood‐brain barrier. To further enhance tumor accumulation, a peptide (T7) that binds to the transferrin receptor on cerebral vascular endothelial cells and thus facilitates blood‐brain barrier penetration was inserted in the membrane by lipid insertion. Beyond CAR T cells, it has also been shown that using membranes derived from TCR T cells has excellent targeting capabilities with tumor retention exceeding two‐fold that of the uncoated and non‐specific membrane‐coated groups [279].

Given their targeting capabilities and the growing interest in using ACT for solid tumors, engineered membrane‐coated NPs have been further developed to enhance ACT, specifically CAR‐T cell therapy, for solid tumors. By utilizing the CAR T cells as the membrane source, NPs can be injected before the CAR T cells to pretreat the tumor to make it a more favorable environment for the CAR T cells, increasing their infiltration and activity in the tumor. In this context, a multifunctional nanocatalyst consisting of anti‐CD19 CAR‐T cell membrane encapsulating Ag_2_S quantum dots and horseradish peroxidase‐loaded polydopamine‐coated gold NPs was developed to enhance CAR‐T cell therapy for the treatment of NALM 6 (human B cell precursor acute lymphoblastic leukemia) solid tumors [285]. Gold NPs are effective photothermal agents and sonosensitizers, allowing the generation of ROS under ultrasound radiation by interacting with surrounding oxygen, thus enabling both sonodynamic therapy and PTT, which was enhanced by polydopamine. The nanocatalyst preferentially accumulated in the tumor in a humanized NALM 6‐tumor‐bearing immunodeficient (NCG) mouse model. Gold NPs possess glucose oxidase‐like and peroxidase‐like activity, enabling the degradation of glucose to produce hydrogen peroxide. Consequently, glycolysis in the tumor cells was inhibited, thereby reducing lactate production and lowering the extracellular acidification rate. This reprogrammed the tumor immunosuppressive microenvironment, making it a more favorable environment for the CAR T cells and increasing their activation in the tumor. Additionally, horseradish peroxidase catalyzed the production of O_2_ from hydrogen peroxide, enhancing the efficiency of sonodynamic therapy and PTT. As a result, under combined ultrasound and NIR laser irradiation, the NPs induced immunogenic cell death of the tumor cells. Combined with CAR‐T cell treatment, tumor growth was inhibited, and a long‐term immune memory effect was observed, which significantly inhibited the tumor recurrence and metastasis development. This led to a high survival rate and a recurrence rate of only 20% after 50 days.

**FIGURE 8:**
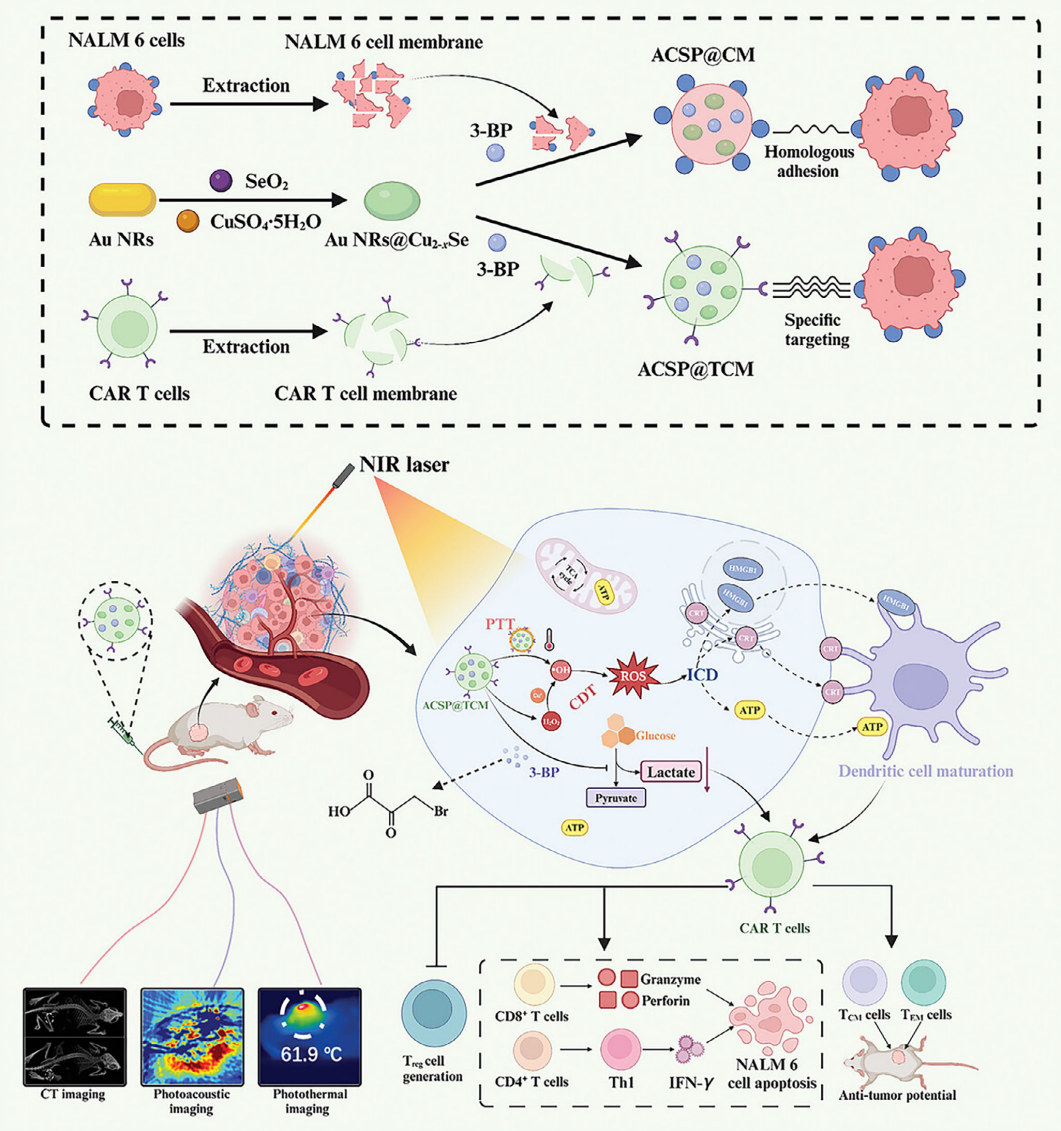
Top) Illustration of the preparation of a nanocatalyst coated with CAR‐T cell membranes (ACSP@CM) and with homologous tumor cell membranes (ACSP@TCM). Bottom) Application in multimodal imaging‐guided synergistic chemodynamic therapy/PTT to enhance CAR‐T cell therapy in solid tumors. CM, TCM, Au NRs, and 3‐BP stand for cell membrane, tumor cell membrane, gold nanorods, and 3‐bromopyruvate, respectively. Reproduced with permission [286]. Copyright 2024, John Wiley and Sons.

The same group developed a different nanocatalyst for chemodynamic therapy, which consisted of CD19‐targeting CAR‐T cell membrane encapsulating core/shell gold nanorods/Cu_2‐x_Se and the drug 3‐bromopyruvate (Figure 8) [286]. While the gold nanorods provided photoacoustic and photothermal properties, Cu_2‐x_Se enabled ROS generation under laser irradiation, causing immunogenic cell death. The drug 3‐bromopyruvate is a glycolysis inhibitor, thus reducing the efflux of lactic acid, which helped remodel the immunosuppressive TME. The nanocatalyst was able to enhance CAR‐T cell therapy for the treatment of solid tumors, inhibiting tumor growth in vivo in NALM 6 tumor‐bearing mice. Interestingly, in this study, the targeting effect of the CAR was also validated by comparing CAR‐T cell membrane‐ and cancer cell (NALM 6) membrane‐coated NPs in vitro. While the CAR provides specific cancer cell targeting, the NALM 6 cell membrane can adhere to cancer cells by homologous adhesion. The results clearly showed an increased targeting of the tumor cells caused by the CAR‐T cell membrane, further demonstrating the potential of engineered cell membrane‐coated NPs to augment ACT.

In summary, the use of engineered cell membrane‐coated NPs in cancer treatment is a highly promising field, due to an increased accumulation in the tumor thanks to the active targeting of the engineered receptors, and prolonged blood circulation time provided by the cell membrane. Alternatively, the intrinsic targeting capability of certain cell membranes can be exploited, and engineered receptors can have therapeutic functions. Preferential tumor accumulation enables targeted drug delivery and NP‐mediated therapies, including hyperthermia, sonodynamic, and chemodynamic therapies. While several studies have shown that engineered cell membrane‐coated NPs can be used for a single therapeutic approach, the technology has also been extended to augment ACT in the treatment of solid tumors. However, as most of the studies have relied on phototherapies, the treatment is limited to rather superficial tumors or tumors accessible by endoscopy, due to the limited light penetration depth of lasers. To broaden clinical applicability, particularly for more deep‐seated tumors, future research in the field should also focus on light‐independent treatments, such as MH, sonodynamic, and chemodynamic therapies. Moreover, thorough evaluation of the stability of cell membrane‐coated NPs in vivo, as well as immune responses and toxicity profiles, must be conducted. Indeed, while some proteins are involved in targeting and immune evasion, many other abundant proteins exhibit interactions that remain poorly understood.

## 7 Safe and Sustainable by Design Principles for Using Nanoparticles in Medicine

While the preceding sections have highlighted the biological mechanisms, clinical promise, and technological sophistication of NP‐enabled ACT, these advances inevitably raise broader questions that extend beyond efficacy and safety at the patient level, which are typically the main priorities when developing a novel treatment. Concerns have been raised that the unique properties of NPs could lead to unintentional effects on human health and the environment [287]. Thorough and rigorous studies are therefore needed to ensure appropriate biocompatibility, biodistribution, and immunogenicity of the NPs, among other aspects, thereby establishing their benefit‐risk profile. However, the design, production, use, and end‐of‐life of NPs also introduce environmental, occupational, and systemic considerations that are seldom addressed in biomedical research but are essential for the responsible translation of these technologies. Embedding principles of safety and sustainability into NP development for medical applications offers a framework to minimize unintended risks while preserving therapeutic innovation. This section, therefore, shifts the focus from biological performance to a life‐cycle‐informed perspective, examining how NPs used in ACT can be developed, assessed, and regulated to ensure not only clinical benefit, but also long‐term environmental and societal sustainability.

**FIGURE 9:**
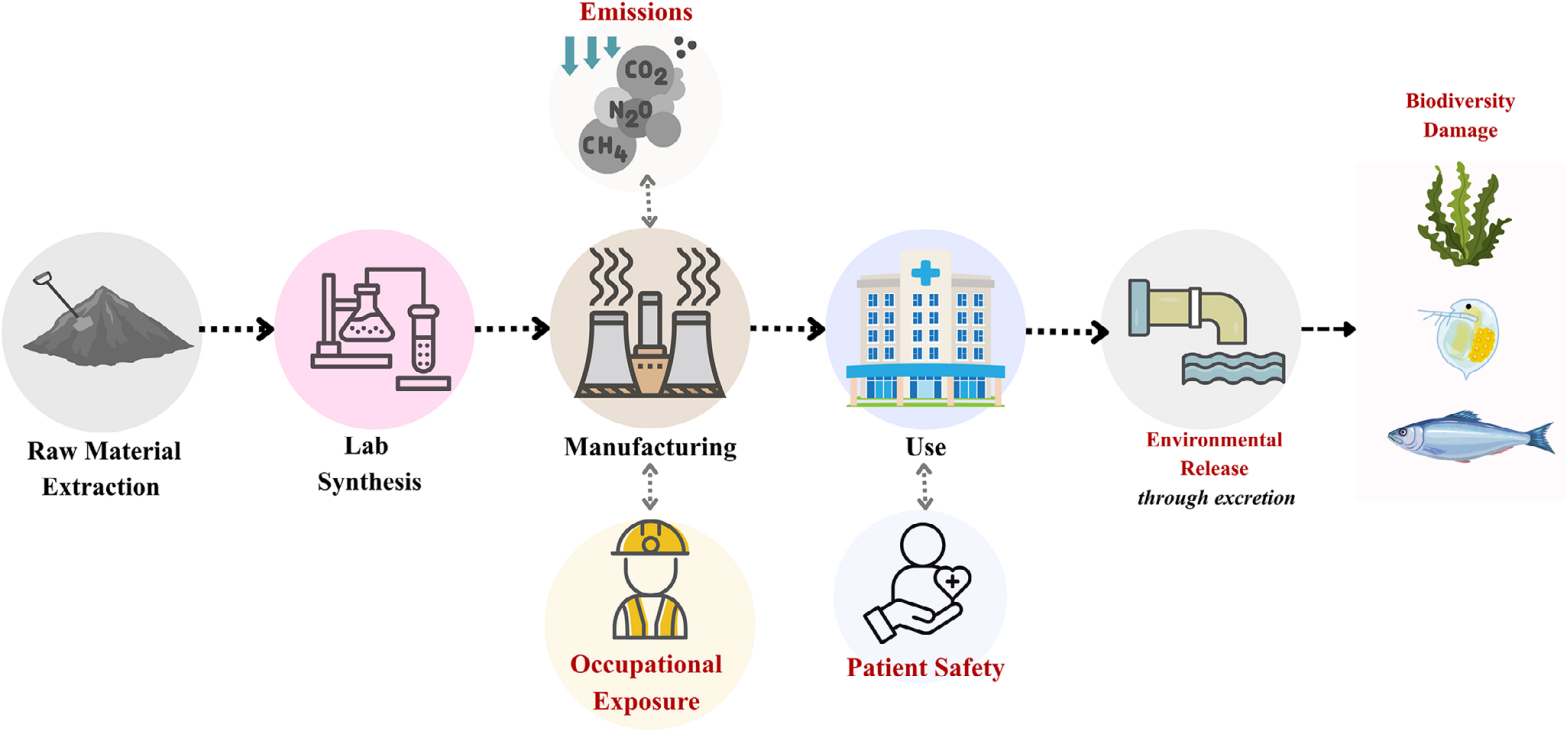
Simplified life cycle of pharmaceuticals and nanomedicines. The hotspots that should be considered during the design phase of a new product are marked in red. Manufacturing, occupational exposure, and use are the main points from which risks and associated hazards emerge, and they are inevitably linked to laboratory synthesis and to the choices made during the early stages.

Figure 9 shows the full life cycle of a nanomedicine with critical steps highlighted where safety and sustainability issues occur. Along the entire life cycle, from laboratory synthesis and manufacturing to clinical use, patient excretion, and final environmental release, significant challenges and measurable impacts on occupational health and the environment emerge at every stage. In the following sections, we will focus on some of the most relevant challenges.

### 7.1 Occupational Exposure

Not only are patients exposed to nanomedicines, but also the workers involved in their synthesis and manufacturing. Workplace exposure to NPs has been well documented, with inhalation identified as the primary route of exposure [288]. A study with 141 workers showed a clear dose‐response relationship between NP levels and inflammatory biomarkers in exhaled breath [289]. Additional evidence links NP exposure to pulmonary dysfunction and inflammation in workers [290]. Peak exposures mainly occur during synthesis, transfer, and cleaning tasks [291, 292]. Consistent with these activity hotspots, systematic reviews and meta‐analyses showed significant increases in oxidative stress biomarkers and pro‐inflammatory cytokines among workers exposed to NPs [293, 294]. Similar exposures have been documented in research laboratories as well [295]. This evidence supports implementing process designs that systematically incorporate worker safety.

### 7.2 Environmental Release, Transformation, and Ecotoxicity

Once administered to patients, nanomedicines are often incompletely metabolized, leading to excretion via urine and feces, entering the wastewater system, where incomplete removal can lead to exposure in different environmental compartments, such as surface water, soil, and sediment [296]. They undergo complex transformations, including aggregation, dissolution, and interaction with natural colloids, as well as the formation of an “eco‐corona” (a layer of biomolecules and organic materials formed when the NPs interact with the environment) [297], altering their bioavailability and mobility [298, 299, 300, 301]. Persistence of nanomedicines in the environment is influenced by their composition. Silver NPs, for example, gradually release Ag^+^ ions, sustaining antimicrobial activity long after their initial transformation [302], while gold NPs exhibit minimal dissolution but accumulate in sediments, raising concerns of long‐term persistence [303, 304, 305].

NP ecotoxicity arises from their unique physicochemical properties, which might induce oxidative stress, membrane damage, and biomolecular interactions in organisms depending on the type of NPs [306]. Their small size facilitates cellular uptake and translocation across biological barriers, potentially leading to bioaccumulation within the organism and biomagnification (i.e., the increase in contaminant concentration at higher trophic levels as predators accumulate substances from the contaminated prey). Meta‐analyses demonstrated that nondissolvable NPs (e.g., TiO_2_, Au, or carbon nanotubes) can accumulate significantly in freshwater organisms [307].

### 7.3 Environmental Sustainability of Nanomedicines

Nanomedicine manufacturing is often complex and requires precise control over numerous parameters, including the use of toxic solvents and reagents. The high level of purity required by the pharmaceutical sector and extensive downstream processing are responsible for high resource and energy demands [308]. In this context, the life cycle assessment methodology provides a structured framework in order to evaluate the overall environmental impacts of a nanomedicine from its raw material extraction to the final disposal, focusing not only on the impacts of the nanomedicine itself but on all materials and energy amounts required in order to produce it [309]. So far, performing robust life cycle assessments for NPs remains difficult, introducing substantial uncertainty and inconsistent results [310, 311]. Various studies confirmed that the environmental profiles of NPs differ substantially depending on the actual production pathway [312, 313]. In conclusion, existing evidence highlights NP production as an intensive process with distinct environmental challenges. Incorporating the evaluation of the environmental impacts early in development is therefore critical to identify impact hotspots and guide innovation towards more sustainable designs.

### 7.4 From Evidence to Action: Operationalizing Safety and Sustainability at the Design Level

A variety of challenges related to occupational, environmental safety, and environmental impact issues can be identified in the life cycle of nanomedicines. However, they should not be treated as constraints that will slow or stop innovation. Instead, the identification of their existence should guide the process of designing a product to reduce the associated risks and impacts. The safe and sustainable by design (SSbD) framework by the European Commission represents a paradigm shift in how novel materials are approached [314, 315, 316]. In alignment with the principles of green chemistry [317, 318], green engineering, and green pharmaceuticals [319], the framework aims to guide the design from the earliest stages of development, combining different methodologies and metrics to assess safety and sustainability aspects without compromising performance. A specific approach for the SSbD assessment of pharmaceuticals has been proposed [320], creating a baseline for its implementation in the nanomedicine field. SSbD also proposes and promotes an approach to assess the socio‐economic sustainability, including economic feasibility, affordability, and stakeholder acceptance.

While this section has focused on the particular safety and sustainability challenges of NPs themselves, the combined use with ACT raises additional issues so far not covered within safety and sustainability assessments, necessitating completely novel approaches how to deal with new biological entities. Besides, the role of the bench scientist, once primarily confined to the design and tests, now has the responsibility to include this perspective in her/his work, being the first brick of the pyramid of innovation. This requires not only a methodological improvement, but also a cultural change in how innovation is conceived, where excellence is measured not solely by performance and efficacy, but also by its capacity to safeguard human health, protect ecosystems, and contribute to a more resilient society.

## 8 Conclusion and Outlook

The rapid advancement of ACT has redefined the landscape of cancer immunotherapy, offering unprecedented therapeutic benefits in hematologic malignancies. Yet, the translation of ACT to solid tumors remains significantly hampered by multiple challenges, most notably the immunosuppressive TME, poor trafficking and infiltration of therapeutic cells, and the risk of severe off‐target or systemic toxicities. To address these limitations, recent breakthroughs in nanomedicine have yielded innovative strategies that harness the unique physicochemical and biological properties of NPs to support and potentiate ACT. A growing body of evidence has demonstrated that NPs can remodel both the physical barriers (dense ECM, abnormal vasculature, and elevated interstitial pressure) and immunosuppressive barriers (presence of Tregs, myeloid‐derived suppressor cells, inhibitory cytokines, and checkpoint signaling) that define the hostile TME. By modifying these compartments, NPs improve adoptive cell infiltration, tumor targeting, directional migration, survival, and ultimately, antitumor activity. One of the most promising NP‐based approaches is NP‐mediated hyperthermia, in which magnetic and/or photothermal NPs generate localized heat under external stimuli. Hyperthermia can not only increase vascular permeability and loosen the tumor ECM, facilitating T cell infiltration, but can also promote the release of damage‐associated molecular patterns and tumor‐associated antigens, thereby enhancing tumor immunogenicity. This dual effect addresses two of the central barriers limiting ACT efficacy in solid tumors: poor accessibility and weak immunostimulation. In parallel, NPs are increasingly investigated as multifunctional carriers for combination therapies. Their high loading capacity, tunable release kinetics, and ability to target specific tissues allow for the codelivery of chemotherapeutics, cytokines, checkpoint inhibitors, or agents used in chemodynamic and photodynamic therapies. By integrating these modalities with ACT, NPs enable synergistic regimens that amplify immune responses while minimizing systemic toxicity. Another important application leverages the magnetic properties of NPs to improve tumor homing of adoptive cells. Under an external magnetic field, magnetic NP‐labeled cells can be guided with high spatial precision toward solid tumors, thereby overcoming one of the most persistent bottlenecks of ACT. Depending on the strategy, NPs can be covalently attached to the cell surface, physically adsorbed, or internalized within cytoplasmic compartments without impairing cellular function. Beyond trafficking and delivery, NPs are also being explored to enhance the in vivo activation, persistence, and expansion of CAR T cells. Traditional CAR‐T cell manufacturing relies on labor‐intensive ex vivo engineering and expansion, which contributes to high costs, long production timelines, and variable patient outcomes. To circumvent these challenges, artificial intelligence (AI)‐driven approaches can improve the precision and efficacy of CAR T cells. For example, AI tools can substantially enhance guide‐RNA design by enabling precise identification of genetic targets and accurate prediction of off‐target effects in CAR T cells [321]. These advances can improve the specificity, safety, and overall therapeutic efficacy of CAR‐T cell therapies. Beyond AI, recent studies have developed CAR‐encoding NPs capable of selectively targeting and transfecting circulating T cells directly in the body of the patient, as shown by promising early clinical trials using lipid and polymeric NPs. This in vivo engineering strategy has the potential to radically simplify the ACT manufacturing pipeline by reducing the need for complex ex vivo cell manipulation, while simultaneously enabling dynamic control over T cell programming. The production of CAR‐functionalized NPs is more cost‐effective and scalable than the autologous and allogeneic cell‐based methods. This could improve scalability and patient access, but major challenges remain. Indeed, in‐depth evaluation of NP pharmacokinetics, in particular targeting specificity, is necessary to avoid unintended programming of multiple types of cells. In addition, NP stability and immunogenicity are unresolved, whereas mRNA‐based CAR expression still lacks durability. AI‐driven design may improve precision, while real‐time monitoring and biomarker‐guided patient selection could increase efficacy. Overall, the convergence of nanomedicine and ACT could lead to a paradigm shift for solid tumor management, which could provide options for patients lacking effective treatments. Indeed, nanotechnology enables the combination of CAR‐T cell therapy with other tumor treatment modalities, such as hyperthermia and chemotherapy, which paves the way for developing personalized medicine. Looking ahead, the progress in NP‐mediated drug delivery, diagnosis, and biosensing will allow the development of precisely targeted therapies tailored to each individual's genetic, molecular, and physiological profile. Integrating nanotechnology with precision medicine and real‐time data analytics will facilitate treatments optimized for maximum efficacy, reduced side effects, and dynamic adaptation to each patient's individual response.

Nonetheless, the integration of both nanomedicine and immunotherapy is still at a nascent stage, as most approaches remain at the preclinical, proof‐of‐concept phase, with substantial challenges to overcome before clinical translation. Clinical trials are necessary to identify the most effective treatment combinations and confirm the long‐term safety and efficacy of personalized strategies. The clinical translation of NP‐based therapies has been constrained in part by safety considerations and variability in pharmacokinetic profiles. To address these challenges, computational approaches and AI‐driven tools, including machine learning, are increasingly being employed to model NP biodistribution and clearance, as well as protein corona composition, to characterize their transport and accumulation within tumor tissues, and to predict therapeutic efficacy across diverse tumor models [322, 323]. Further research is also needed to develop NPs that evade rapid clearance by filtering organs and exhibit high specificity for tumor tissue. Strategies include conjugating targeting ligands, coating NPs with cell membranes to enable immune evasion and homologous targeting, and employing a magnetic field for tumor targeting. Moreover, further mechanistic studies are needed to elucidate the synergy between NP‐mediated hyperthermia and ACT, particularly the underlying immunomodulatory mechanisms. In addition, the relationship between thermal doses and specific immune responses requires further elucidation, necessitating improvements in temperature monitoring. Future research should focus on the development of next‐generation NPs with “sense‐decision” capabilities to allow closed‐loop regulatory strategies. In this regard, nanozyme‐based immunotherapy approaches have been reported, in which catalytic NPs (nanozymes) sense and dynamically modulate the TME through feedback mechanisms to enhance immune activation [324]. Nanozymes are emerging oncological tools that mimic natural enzymes with high catalytic stability. Owing to their multi‐enzyme activities and TME responsiveness, they can synergize with immunotherapy, PTT, photodynamic therapy, and chemodynamic therapy to improve tumor eradication. They can selectively reprogram the TME to stimulate immune responses, remodel metabolism, and establish self‐amplifying therapeutic loops, including dendritic cell maturation, T cell activation, and cytokine release. They integrate external physical cues (e.g., light or magnetic field) or intrinsic biochemical signals, such as pH or enzymatic activity, into “sense‐decide‐act” regulatory circuits, enabling closed‐loop and adaptive control of tumor therapy. Engineering point‐of‐care NP manufacturing systems must also be considered to enable rapid production of clinically relevant NPs with reproducible quality at the time and place of need, thereby reducing costs and improving accessibility. As an example, organic NPs, such as liposomes, polymer NPs, and LNPs, encapsulating RNA have been synthesized at the point of care [325]. This could make nanomedicines more broadly accessible and ultimately lead to personalized ACT for patients and improve the overall effectiveness of treatments. Nevertheless, major challenges still remain when using NPs, including regulatory approval, sustainability, scalability, and cost‐effective production. Compared with traditional medicines, nanomedicines demand additional regulatory and developmental attention. In particular, comprehensive long‐term biosafety and ecotoxicological evaluations are indispensable to ensure safe and sustainable therapeutic applications while minimizing environmental impact. Continued research efforts are vital to overcome these hurdles and enable the successful integration of engineered T cells with NP‐based therapies in cancer treatment. An immediate priority for researchers is to pursue innovative, high‐impact studies aimed at identifying the most effective combinations to enhance ACT efficacy against solid tumors, achieve major breakthroughs, and accelerate translation to the clinic. Beyond these advancements, scientists have the responsibility to integrate SSbD principles into their work. This demands not only methodological improvements, but also a cultural shift in how innovation is envisioned, where excellence is defined not solely by performance and efficacy, but also by the ability to protect human health, preserve ecosystems, and foster a more resilient and equitable society.

Key insights:

- NPs can remodel both the physical barriers and immunosuppressive barriers of the TME, for instance, by hyperthermia, thus improving tumor targeting, adoptive cell infiltration, and antitumor activity.
- Under an external magnetic field, NP‐labeled cells can be guided with high spatial precision towards solid tumors.
- Engineered cell membrane‐coated NPs can enhance tumor targeting.
- NPs can be exploited for the codelivery of chemotherapeutics, cytokines, checkpoint inhibitors, or agents used in chemodynamic and photodynamic therapies, resulting in immune response amplification with minimized systemic toxicity.
- CAR‐encoding NPs are capable of selectively targeting and transfecting circulating T cells directly in the body.
- Incorporating safety and sustainability principles into NP development for biomedical applications provides a framework to mitigate unintended risks while maintaining therapeutic innovation.

Outstanding challenges:

- In‐depth evaluation of pharmacokinetics, in particular targeting specificity, long‐term toxicology studies, potential immunogenicity, and biodegradation/biopersistence of NPs;
- Evaluation of the potential biological effects arising from NP loading in T cells;
- Modeling of NP biodistribution and clearance, and prediction of therapeutic efficacy across diverse tumor models by computational approaches and AI‐driven tools;
- Incorporation of SSbD principles with comprehensive safety assessments and ecotoxicological evaluations;
- Development of NPs (e.g., nanozymes) that can sense and dynamically modulate the TME through feedback mechanisms to enhance immune activation;
- Elucidation of the synergy between NP‐mediated hyperthermia and ACT (e.g., immunomodulatory mechanisms);
- Addressing the challenges in vascular normalization approaches related to transient effects and heterogeneity in vessel response;
- Minimization of off‐target tissue damage and variable efficacy across tumor types in enzymatic ECM remodeling strategies;
- Combination of ACT with NP‐assisted metabolic TME reprogramming;
- Improvement of mRNA‐based CAR expression durability mediated by NPs;
- Use of more complex and relevant mouse models, such as orthotopic and/or immunocompetent models;
- Integration of engineered exosomes with CAR‐T cell therapy;
- Integration of nanotechnology with precision medicine and real‐time data analytics for dynamic adaptation to each patient's individual response;
- Performing clinical trials to confirm efficacy and long‐term biosafety of combined ACT and NP‐mediated therapeutic strategies;
- Addressing regulatory approval.

## Conflicts of Interest

The authors declare no conflicts of interest.

## Data Availability

The authors have nothing to report.
